# A mutation in the viral sensor 2’-5’-oligoadenylate synthetase 2 causes failure of lactation

**DOI:** 10.1371/journal.pgen.1007072

**Published:** 2017-11-08

**Authors:** Samantha R. Oakes, David Gallego-Ortega, Prudence M. Stanford, Simon Junankar, Wendy Wing Yee Au, Zoya Kikhtyak, Anita von Korff, Claudio M. Sergio, Andrew M. K. Law, Lesley E. Castillo, Stephanie L. Allerdice, Adelaide I. J. Young, Catherine Piggin, Belinda Whittle, Edward Bertram, Matthew J. Naylor, Daniel L. Roden, Jesse Donovan, Alexei Korennykh, Christopher C. Goodnow, Moira K. O’Bryan, Christopher J. Ormandy

**Affiliations:** 1 Garvan Institute of Medical Research and The Kinghorn Cancer Centre, Darlinghurst, NSW, Australia; 2 St. Vincent’s Clinical School, UNSW Medicine, UNSW Sydney, NSW, Australia; 3 Australian Phenomics Facility, The Australian National University, Canberra, ACT, Australia; 4 School of Medical Sciences and Bosch Institute, Sydney Medical School, University of Sydney, Sydney, NSW, Australia; 5 Department of Molecular Biology, Princeton University, Princeton, New Jersey, United States of America; 6 The School of Biological Sciences, Monash University, Clayton, Australia; University of Melbourne, AUSTRALIA

## Abstract

We identified a non-synonymous mutation in *Oas2* (I405N), a sensor of viral double-stranded RNA, from an ENU-mutagenesis screen designed to discover new genes involved in mammary development. The mutation caused *post-partum* failure of lactation in healthy mice with otherwise normally developed mammary glands, characterized by greatly reduced milk protein synthesis coupled with epithelial cell death, inhibition of proliferation and a robust interferon response. Expression of mutant but not wild type *Oas2* in cultured HC-11 or T47D mammary cells recapitulated the phenotypic and transcriptional effects observed in the mouse. The mutation activates the OAS2 pathway, demonstrated by a 34-fold increase in RNase L activity, and its effects were dependent on expression of RNase L and IRF7, proximal and distal pathway members. This is the first report of a viral recognition pathway regulating lactation.

## Author summary

Using ENU-mutagenesis in mice we discovered a pedigree with lactation failure. Mammary development through puberty and pregnancy appeared normal in mutant animals, but the activation of lactation failed in the immediate post partum period and no milk reached the pups. Failure of lactation was accompanied by greatly diminished milk protein synthesis, decreased epithelial cell proliferation, increased epithelial cell death and a robust interferon response. A non-synonymous mutation in *Oas2* (I405N) in the viral sensor Oas2 was found and expression of mutant *Oas2* in mammary cells recapitulated these phenotypes. RNase L, the most proximal effector of OAS2 action, was activated in the mammary glands of mutant mice and in mammary cells expressing mutant Oas2. Knockdown of RNase L, or the distal pathway member IRF7, prevented these effects, indicating that the mutation in OAS2 caused activation of the viral signaling pathway. These results show that viral detection in the mammary gland can prevent lactation.

## Introduction

The oligoadenylate synthetase (OAS) enzymes are activated by detection of double stranded RNA produced during the viral life cycle, and in response polymerize ATP into 2´-5´ linked oligoadenylates (2-5A) of various lengths. The 2-5As then bind and activate latent RNase L, which degrades viral and host single stranded RNAs, so disrupting the viral life cycle [1]. It has been reported that OAS1 has antiviral activity independently of RNAse L [2], that OAS2 binds to NOD2 [3], and that OASL binds RIG-I [4], pointing to additional mechanisms of action. Although mechanistic detail is lacking, it is proposed that OAS enzymes can activate anti viral responses via mechanisms independently of 2-5A production, by direct interactions within the viral signaling complex. For example, this complex is tethered to the mitochondrial outer membrane by the scaffold protein MAVS, and contains RIG-I, related helicase MDA5, and possibly OAS family members [5]. OAS family members may also mediate apoptosis outside the context of viral infection [6,7]. Here we report a mutation of OAS2 that produces lactation failure in an otherwise normal mouse. This is the first demonstration that a viral recognition pathway can regulate lactation.

## Results

**Fig 1:**
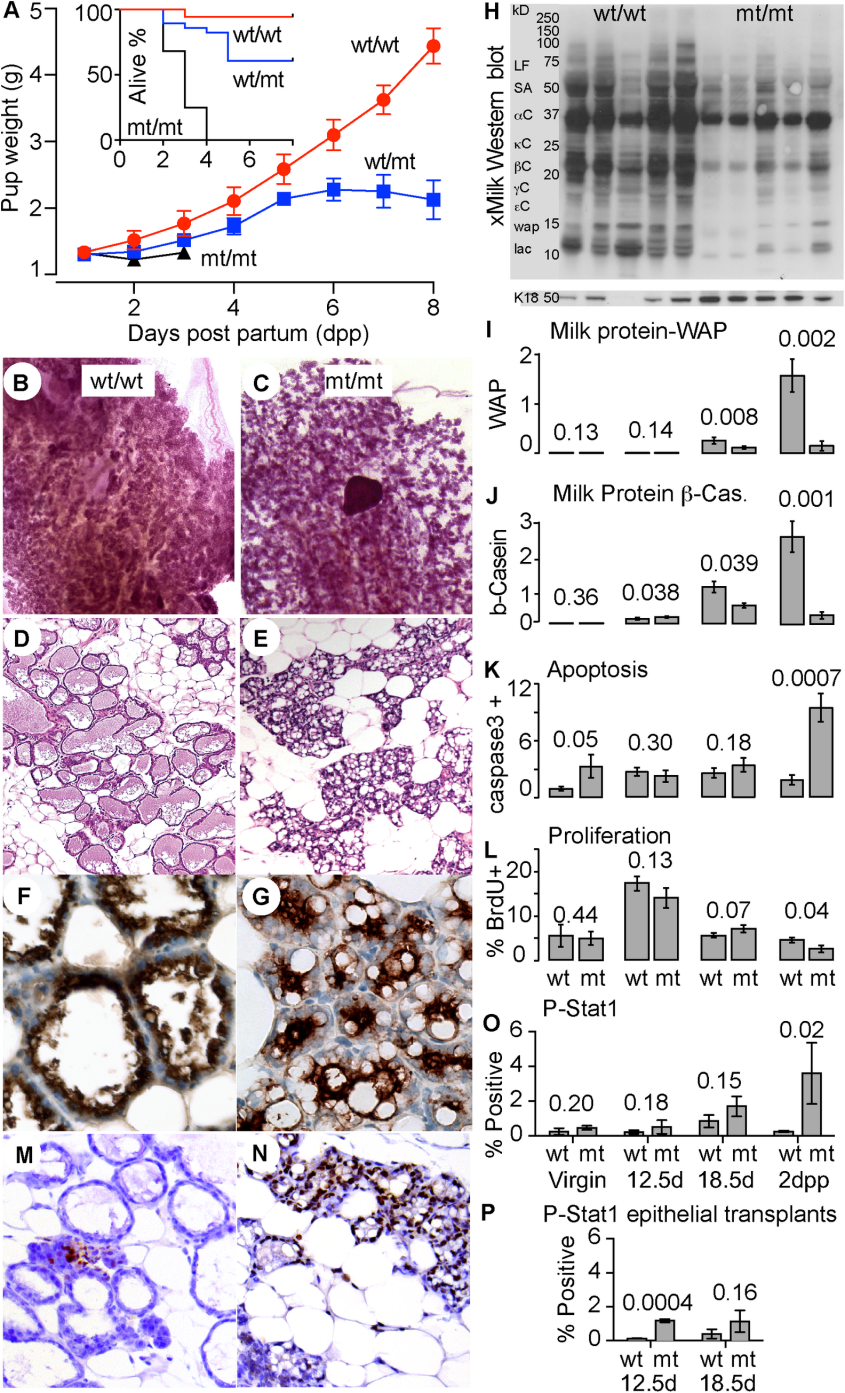
Discovery of a pedigree with dominant inheritance of failed lactation. **(A)** Lactation performance of dams of the indicated genotypes (wild type; wt/wt mutant; mt/mt) assessed by pup weight-gain or survival (inset). Error bars show standard error of the mean for 4–5 litters per genotype of 7 pups each. wt/wt n = 35, wt/mt n = 28 and mt/mt n = 28 pups. **(B and C)** Whole mount histology of the 4^th^ inguinal mammary gland showing lobuloalveolar development at 2 days post partum (dpp) in wt/wt or mt/mt mice. **(D and E)** Corresponding haematoxylin-eosin histochemistry. **(F and G)** Corresponding immunohistochemistry for milk protein expression. **(H)** Corresponding Western blot for milk proteins. Molecular size is shown together with the established sizes of the indicated milk proteins [35]. Lactoferrin (LF), serum albumin (SA), caseins α,κ,β,γ and ε, whey acidic protein (wap) and alpha lactalbumin (lac). **(I)** Quantification of *Wap* mRNA by qPCR at in wt/wt or mt/mt mice. **(J)** Quantification of β-casein (β-Cas) mRNA by qPCR. **(K)** Quantification of epithelial cell death by immunohistochemistry for cleaved caspase 3, results are the number of positively stained epithelial cells as a percentage as a percentage of total number of epithelial cells per field. **(L**) Quantification of epithelial cell proliferation by incorporated BrdU expressed as a percentage of total number of epithelial cells per field. **(M and N**) immunohistochemistry for phosphorylated (P) STAT1 at 2 days post partum (dpp) in wt/wt or mt/mt mice. **(O)** quantification of P-STAT1 in wt/wt or mt/mt mice by immunohistochemistry, results are the number of positively stained epithelial cells as a percentage of total epithelial area. **(P)** Quantification of P-STAT1 in wt/wt or mt/mt mammary transplants by immunohistochemistry, results are the number of positively stained epithelial cells as a percentage of total epithelial area. **(I-J and O)** wt/wt n = 4–5 mice, mt/mt n = 3–5 mice per time point **(P)** wt/wt n = 3–5 mice, mt/mt n = 2–5 per time point. Student’s t-test p values are given, error bars are standard error of the mean.

Using N-ethyl-N-nitrosourea (ENU) mutagenesis and a screen for failed lactation we established a mouse line in which heterozygous (wt/mt) dams showed partial penetrance of poor lactation, producing litters that failed to thrive, while homozygous (mt/mt) dams experienced complete failure of lactation (Fig 1A), providing a dominant pattern of inheritance. Development of the mammary ductal network during puberty, and of the lobulo-alveolar units during pregnancy, was normal in mt/mt dams (S1A and S1B Fig). The onset of milk protein synthesis also showed no defects during pregnancy by immunohistochemistry or western blot (S1C Fig and S1D Fig). Lactation failure in mt/mt mice at 2 days *post-partum* (2dpp) was seen as failure of alveolar expansion and retention of lipid droplets and colostrum (Fig 1B–1G). Western blotting for milk (Fig 1H and S1C and S1D Fig) showed greatly reduced expression of all the major milk components at 2dpp relative to the level of the epithelial cell marker cytokeratin 18. Quantitative PCR for the mRNAs for the milk proteins whey acidic protein (WAP) and β-casein (β-Cas) showed reduced levels in mt/mt dams (mt) compared to wt/wt dams (wt) at 18 days *post-coitus* (dpc) and especially at 2dpp (Fig 1I and 1J). The number of cleaved Caspase-3 positive epithelial cells increased (Fig 1K) and BrdU incorporation by the epithelium was reduced, indicating increased cell death rate and decreased cell proliferation respectively (Fig 1L).

We used immunohistochemistry to examine STAT1 activation as it is an interferon regulated gene involved in mammary gland involution [8]. In wt/wt dams at d18.5 of pregnancy and 2 days post partum, we observed scattered regions of phosphorylated Stat1 staining in tightly packed areas of small and unexpanded alveoli (Fig 1M). These regions were very rare at the other stages of development examined. In mt/mt animals Stat1 phosphorylation was again seen within regions of small unexpanded and tightly packed alveoli (Fig 1N), but at day 18.5 of pregnancy, these regions of STAT1 phosphorylation occurred at a far greater frequency than in wt/wt glands, and instead of receding in the post partum period like wt/wt glands, the frequency of this pattern of staining increased further (Fig 1O). We examined Stat1 phosphorylation in mammary glands formed by transplant of epithelium from mt/mt or wt/wt animals into the mammary fat pads of prepubescent wild type mice cleared of endogenous epithelium. We again observed a statistically significant increase in Stat1 phosphorylation in mt/mt transplants in the pre-partum period (transplants can’t interrogate the post partum period), demonstrating that the ENU-mutation operates autonomously via the mammary epithelial cell (Fig 1P).

**Fig 2:**
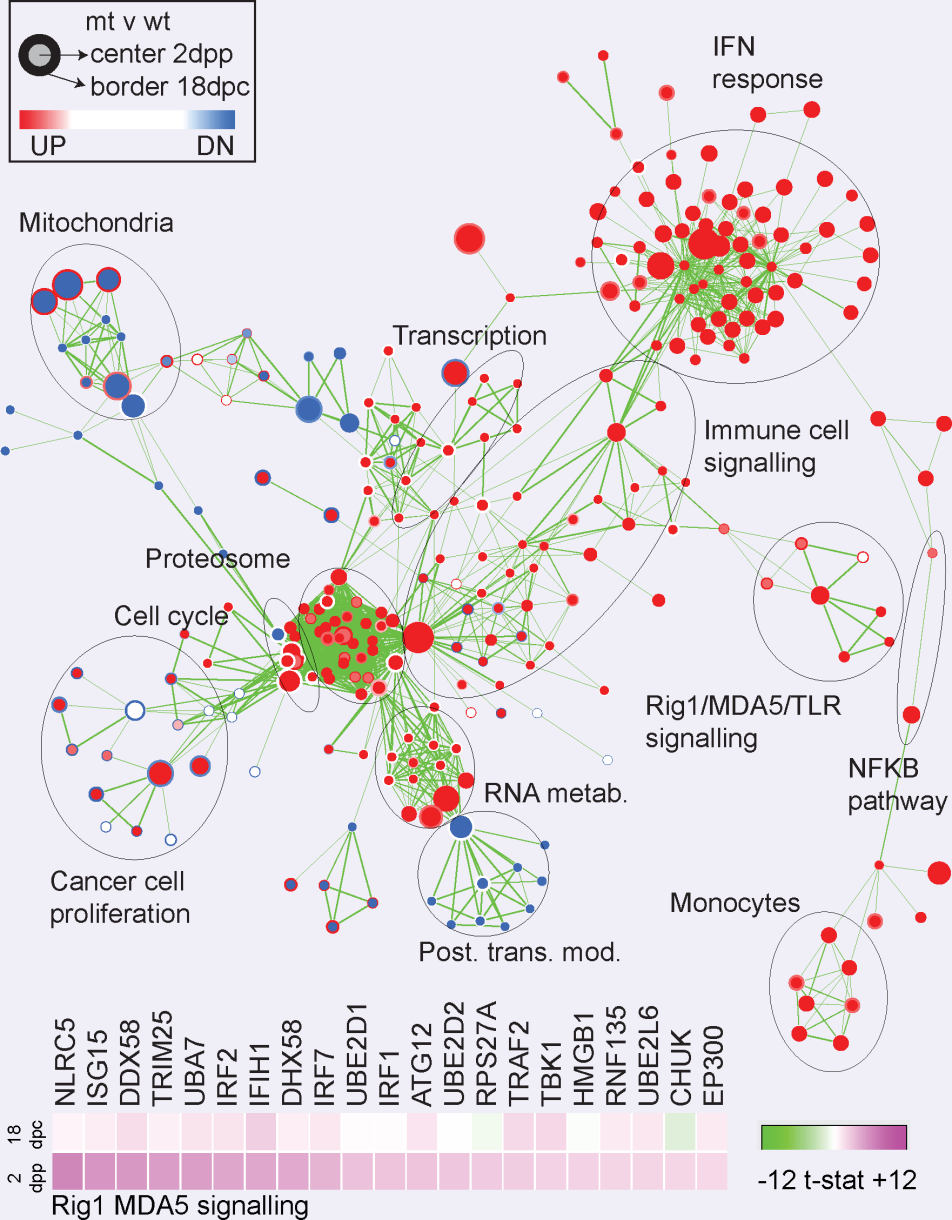
Effects of OAS2 mutation on global patterns of gene expression in the mammary gland. Whole mouse mammary glands from homozygous *Oas2* mutant (mt) or wild type (wt) animals were profiled using Affymetrix MTA arrays. Differential gene expression was ranked by the limma t-statistic and this was used as the input for gene set enrichment analysis to identify functional signatures. The enrichment-map plug in for Cytoscape was used to visualize the results. Each node represents a gene set and the expression of genes comprising the leading edge of some of these sets is shown as heat maps of the t-statistic. Labels indicate the function of the clustered gene sets. Gene expression in mt animals is compared with wt animals at 2dpp (node center color) or 18dpc (node edge color). Red indicates enrichment of expression the gene set and blue suppression of expression.

We used the Affymetrix Mouse Transcriptome Assay (MTA) 1.0 GeneChip to measure changes in gene expression underlying these events. We profiled RNA transcripts in the mammary glands at 18dpc and 2dpp from wt/wt and mt/mt mice. A Gene Set Enrichment Analysis (GSEA) of genes was carried out using the Limma t-statistic as a measure of ranked differential expression and visualized with the Enrichment Map plugin for Cytoscape. We compared gene expression changes between mt/mt and wt/wt mice at 18dpc or 2dpp (Fig 2, shown in detail S2 Fig). This identified a robust enrichment of a prominent cluster of gene sets involved in the interferon response in postpartum mt/mt but not wt/wt mammary glands, which increased in magnitude between 18dpc and 2dpp. Genes in these sets included the interferon-induced genes *Isg15*, *Mx1*, *Rsad2*, *Oas1*, *Oas2 and OasL1*. Interferon-induced genes involved in the molecular pattern response pathway were also induced, such as *Ddx58* (RIG-1), *Dhx58* (RIG-1 regulator), *Mavs* and *Nlrc5* (NOD5). Additional downstream transcriptional regulators of the interferon response, such as *Stat1*, *Irf7* and *Irf9*, were upregulated. In mt/mt glands this was accompanied by increased expression of a broad range of mitochondria-associated cell death genes such as *Tnsfs10* (TRAIL), *Acin1*, *Birc2*, *Traf2*, *Bcl2l1* (BCL-XL), *Bcl2l11* (BIM), *Apaf1*, *Dffb*, *Xaf and Ripk1*. Very similar results were obtained using an independent analysis technique based on self-organizing maps (S3 Fig). These results are also presented as a.txt table (S1 Table) of the 5000 probes showing most-changed expression. This transcriptional data indicates that a robust interferon response is induced by the mutation.

**Fig 3:**
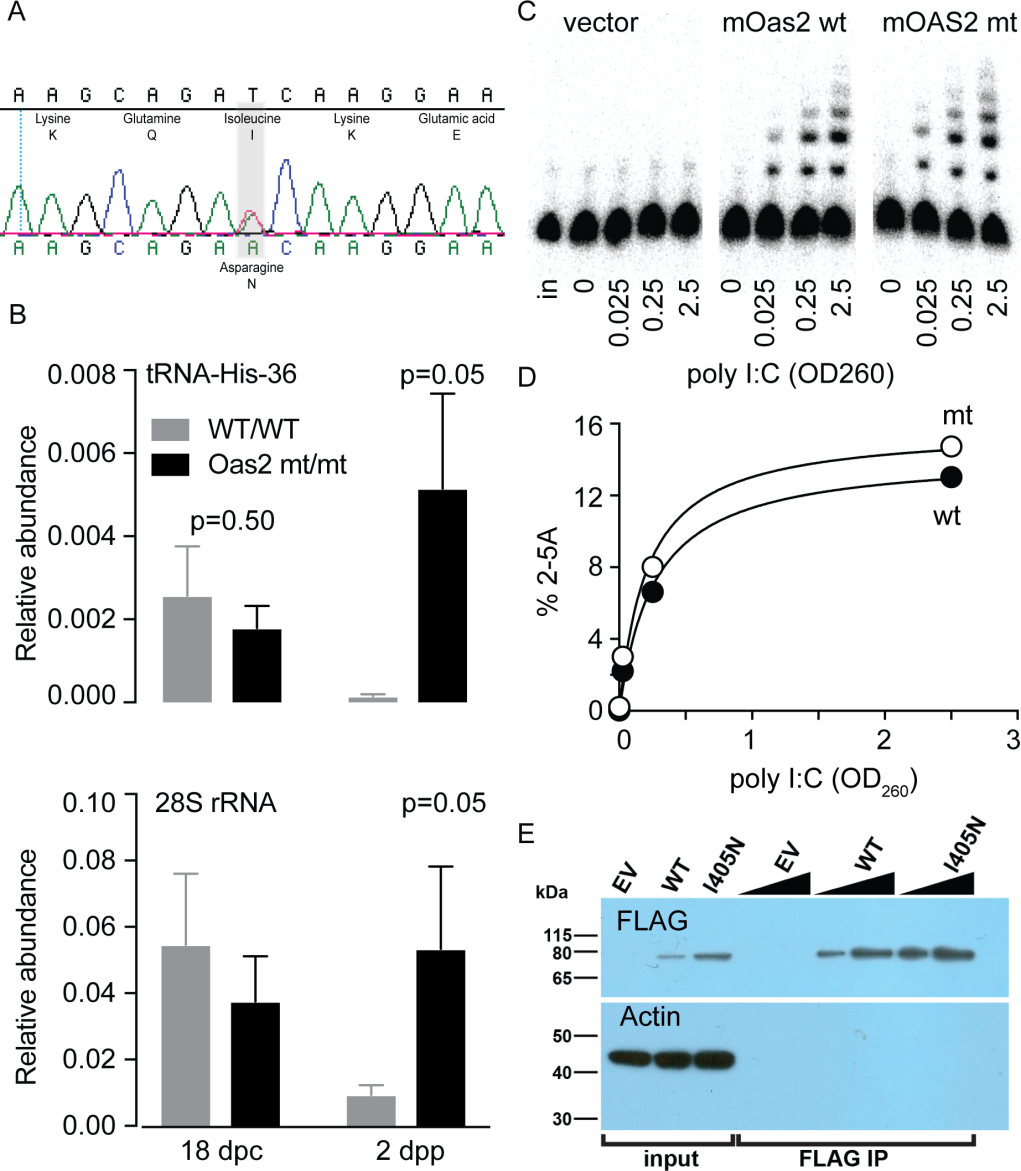
Enzymatic properties of mutant OAS2. **(A**) Details of the mutation in *Oas2* showing the ENU-induced SNP changing isoleucine to asparagine. **(B)** RNAseL activity measured as the abundance of RNase L-specific cleavage of tRNA-His-36 (upper panel) or rRNA (lower panel) at day 18 of pregnancy (d18pc) and two days post partum (2dpp). **(C**) Representative denaturing PAGE separating 2-5A species of different molecular weights synthesized in a cell free system by mutant (mt) or wild type (wt) mouse OAS2, in response to activation by different concentrations of the double-stranded RNA mimic polyI:C. **(D**) quantification of the data in panel C. **(E**) western blot demonstrating similar OAS2 protein input to the assay above.

PCR genotyping of polymorphic markers and their co-inheritance with lactation failure narrowed the mutation to a 4Mb region of chromosome 5 between rs3662655 and rs2020515. We expected 4–8 ENU mutations per 4Mb and our strategy was to sequentially sequence exomes and then to experimentally validate when an exonic mutation was discovered. Sequencing revealed a T to A base change in *Oas2*, resulting in a non-conservative isoleucine to asparagine amino acid substitution (I405N; Fig 3A and S4A Fig) in a conserved region of the OAS2 catalytic domain (S4B and S4C Fig). In wt/wt animals *Oas2* was expressed at a relatively low level until the establishment of lactation, when the level of *Oas2* mRNA increased by 20 fold (S5A Fig) and subsequently fell during early involution. Changes in *Oas2* expression in wt/wt animals compared to mt/mt animals are shown in S1E Fig. Using immunohistochemistry we observed corresponding changes in OAS2 levels in the mammary epithelium (S5B Fig).

We measured RNase L activity in the mammary glands of wt/wt and mt/mt mice at 18 days post coitus (dpc) and 2 days post partum (dpp) using a recently developed technique [9]. In wt/wt mice we observed a fall in RNase L activity from pre lactation at 18 dpp to lactation at 2 dpp despite the rise in OAS2 over this period (Fig 3B top panel). In contrast mt/mt animals showed an increase in RNase L activity over this period, so that at 2 dpp, RNase L activity was 34 fold higher in mt/mt animals. PCR for RNase L-cleaved rRNA showed a six-fold increase RNase L activity (Fig 3B lower panel), while non-RNase L generated cleavage was negligible. Bioanalyzer profiles of RNA (S5C Fig) showed increased RNA degradation in mt/mt animals, but not to the extent that appreciable loss of the 18S or 28S ribosomes was seen, and which may be a result of both RNase L dependent and independent mechanisms. Although robust activation of RNase L can cause the loss of the 18S and 28S ribosomes [10], recent findings show that ribosomal degradation is not required for RNase L to stop protein synthesis [9]. To determine if the mutation altered OAS2 enzyme activity we purified the mutant and wild type forms of mouse OAS2 expressed in HeLa cells by FLAG-immunoprecipitation. Using a cell-free system we observed that both mutant and wild type forms of OAS2 showed induction of enzyme activity by the double-stranded RNA mimic poly (I:C), seen as the formation of a series of 2-5A species resolved by denaturing PAGE. Both mutant and wild type forms of OAS2 showed similar sensitivity to increasing poly (I:C) concentrations (Fig 3C, quantified in Fig 3D). Western blotting showed that the immunoprecipitates used in these experiments had similar OAS2 levels (Fig 3E). These experiments show that the ENU-induced mutation in OAS2 does not change the size range of oligoadenylates that it produces, its capacity for 2-5A synthesis, or its sensitivity to activation by poly (I:C). This assay uses a cell free system, so we cannot exclude a mechanism where mutant OAS2 activates RNase L activity via an indirect effect to increase the active 2-5A pool without altering its rate of synthesis, such as reduced 2-5A depletion or loss of 2-5A sequestration. Another possibility is that mutant OAS2 has an altered molecular interaction with a species that increases its enzymatic activity, but which is lost in the immunoprecipitation of OAS2 in this assay. Regardless, the mutation in *Oas2* activates RNase L in mice and tissue culture models.

**Fig 4:**
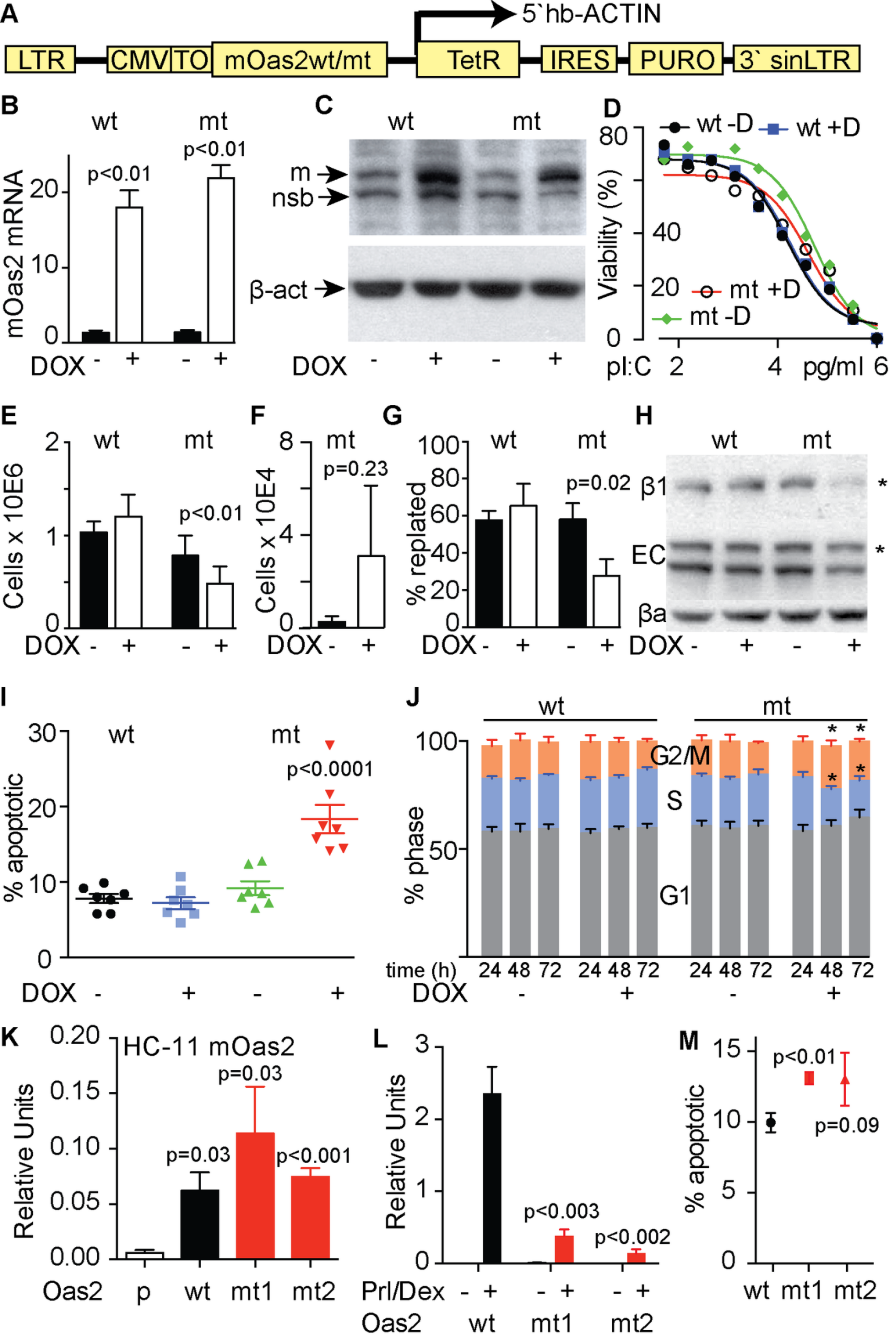
The effects of inducible expression of mutant and wild type *Oas2* in T47D cells. **(A**) pHUSH ProEx expression vector used to express either mutant (mt) or wild type (wt) mouse *Oas2* in T47D cells in response to doxycycline (DOX). **(B**) relative expression of mt and wt *Oas2*. **(C**) Western blot showing induction of mouse OAS2 (m) running just below endogenous human OAS2 protein, with both bands above a non-specific band (nsb). **(D**) Sensitivity of the cells lines to poly I:C (pl:C) with and without DOX induction of mt and wt *Oas2*. **(E**) Effect of mt and wt *Oas2* on adherent cell number after 72h. (**F**) Cell detachment (numbers of live cells in supernatant fraction) caused by mt *Oas2*. **(G**) Effects of mt or wt *Oas2* on replating of T47D cells in a 4 hour trypsin only replating assay after 48h of DOX. **(H**) Expression of β1 integrin (β1), E-cadherin (EC) and β-actin (βa) in response to induction of mt or wt *Oas2*. **(I**) apoptotic response to induction of mt or wt *Oas2*. Data represents the average of 7 independent experiments. **(J**) cell-cycle-phase distribution at the indicated times following induction of mt or wt *Oas2*. Data represents the average of 5 independent experiments. *p<0.01. ANOVA 4I and J. **(K)** *Oas2* expression in parental (p) normal mouse mammary HC11 cells or in cells constitutively expressing mt or wt *Oas2*. **(L)** Effect of wt or mt Oas2 on beta Casein in HC11s after 72 hours of prolactin (Prl) and Dexamethasone (Dex) stimulation. **(M)** Effect of mt or wt *Oas2* expression on cell death at 96 hours in HC11 cells after transient transfection. All data are representative of 3 independent experiments in response to 72h of DOX except otherwise specified. Paired t-tests 4B,E,F, G, L and M.

We constructed a model of doxycycline (Dox)-inducible expression of mutant or wild type *Oas2* in T47D human breast cancer cells (Fig 4A). These models produced a 20-fold induction of *Oas2* expression (Fig 4B). Western analysis showed the appearance of mouse OAS2 protein following Dox administration just below endogenous human OAS2, both above a non-specific band (Fig 4C). Thus although PCR showed a small amount of leakiness in this system it seems negligible by western blot. Cells expressing either mutant or wild type *Oas2* showed a similar sensitivity to poly (I:C) that was not changed significantly by induction with Dox (Fig 4D). Induction of mutant, but not wild type *Oas2* for 72 h reduced cell number (Fig 4E). Increased cell detachment was observed, but the magnitude of this effect was highly variable between experiments using mutant cells and so did not reach statistical significance at p<0.05 (Fig 4F). Reduced re-plating efficiency however following trypsinization was significant, indicting that cell surface re-expression of adhesion molecules following their trypsin digestion was impaired (Fig 4G). Western analysis of two of these molecules, Beta-1 Integrin (ß1) and E-Cadherin (EC), showed reduced expression in response to Dox-induction of mutant *Oas2*, especially for Beta-1 Integrin, shown in the far right hand side lane (Fig 4H). We used flow cytometry to simultaneously measure cell viability by propidium iodide exclusion and cell death by cell surface expression of Annexin V, in response to *Oas2* expression. While induction of wild type *Oas2* expression produced no apoptotic response, induction of mutant *Oas2* produced a doubling in the number early apoptotic cells within the cultures (Fig 4I). Induction of wild type *Oas2* did not alter the distribution of cells among the phases of the cell cycle, while induction of the mutant produced a shift of cells out of S-phase and into G1 (Fig 4J). Thus the effects of mutant *Oas2* expression in T47D cells reproduce the phenotypes of cell death and reduced cell proliferation seen in the mouse, and indicate that epithelial cell adhesion may also be affected.

Mouse HC-11 cells express milk proteins in response to withdrawal of EGF and the addition of prolactin and dexamethasone, providing a way to examine the effects of mutant and wild type *Oas2* expression on milk protein expression. The inducible vector system used successfully in T47D cells (Fig 4A) proved to be very leaky in HC-11 cells, resulting in high baseline expression of *Oas2* in the pooled clones without DOX treatment. Cloning, in an attempt to find cells without leaky expression, was unsuccessful, but resulted in cell lines with similar levels of constitutive expression of mutant or wild type *Oas2* that was many fold greater than seen in untransfected cells (Fig 4K). Treatment with prolactin and dexamethasone induced beta casein levels in the cell line expressing wild type *Oas2*, and this effect was comparable in magnitude to that seen in parental HC-11 cells, but in the two lines expressing mutant *Oas2* the induction of beta casein was greatly reduced (Fig 4L), reproducing the suppression of milk protein synthesis seen in the ENU-mutant mouse. Transient expression of wild type and mutant *Oas2* in HC11 cells also showed an increase in the basal rate of cell death, reproducing the cell death phenotype (Fig 4M).

We used Affymetrix Human Transcriptome Assay 2.0 GeneChips to profile the changes in gene expression that occurred in T47D cells when either wild type or mutant *Oas2* was induced for 72h, presented as GSEA/Cytoscape (S6 Fig), self organizing maps (S7 Fig) and as table containing the top 500 differentially expressed genes (S2 Table). We compared the transcriptional effects of mutant *Oas2* in T47D cells to the effects in the ENU mouse shown in Fig 2 using Cytoscape (S8 Fig), or self-organizing maps (S7 Fig). The transcriptional effects of mutant OAS2 in T47D cells were very similar to those observed in the ENU-mutant mouse, with the interferon response most prominent. This demonstrates that expression of mutant but not wild type *Oas2* in T47D cells reproduces the molecular phenotypes observed in the ENU mutant mice. While the phenotype in mice is likely to involve additional cells of the immune system, these effects in T47D cells show that the transcriptional phenotype can be elicited via the innate immune response of the mammary epithelial cell, in agreement with the findings made using transplanted ENU-mutant mammary epithelium into wild type mice (Fig 1P).

**Fig 5:**
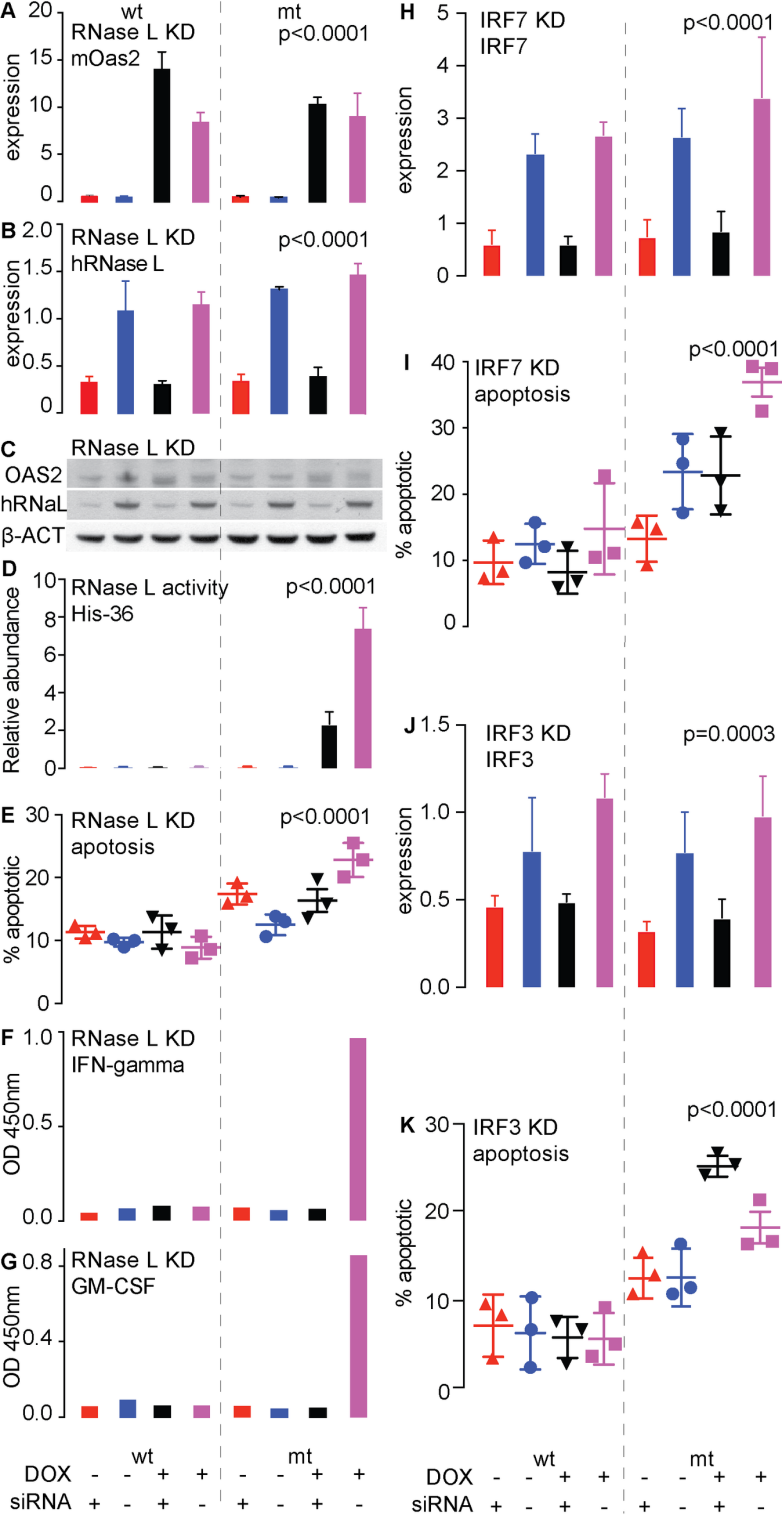
Effects of knockdown of RNASEL, IRF7 and IRF3 on the effects of inducible expression of either mutant (mt) or wild type (wt) mouse *Oas2* in T47D cells. **(A-G)** Provide the context of RNase L knockdown. **(A-C**) Demonstration of Doxycycline (DOX)-inducible expression of wt or mt *Oas2* in T47D cells, and effective knock-down of RNASEL (RNaL) in mt or wt expressing T47D cells by quantitative PCR (**B**) or western blot (**C**). **(D)** Effect of the induction of mt or wt OAS2 on RNase L activity **(E)** Effects of induction of mt and wt *Oas2* expression on apoptosis. **(F)** Effects of these treatments on interferon gamma protein production. **(G**) effects of these treatments on GM-CSF production. **(H**) Demonstration of effective knockdown of IRF7. **(I)** Effects of knockdown of IRF7 on mutant or wild type *Oas2*-driven apoptosis. **(J)** Demonstration of knockdown of IRF3. **(K)** Effects of knockdown of IRF3 on mutant or wild type *Oas2*-driven apoptosis.

OAS2 activates RNaseL. In T47D cells we used siRNA against human *RNASEL* to knockdown its expression in the context of Dox-induction of mutant or wild type mouse *Oas2*. In these experiments the induction of *Oas2* in response to Dox was robust and knockdown of *RNASEL* was very effective, as demonstrated by qPCR (Fig 5A and 5B) and by western blot (Fig 5C). Induction of wild type *Oas2* had no effect on RNase L activity, cell death or cytokine levels and knockdown of RNASEL was without consequence to these endpoints. In contrast, induction of mutant *Oas2* produced a large increase in RNase L activity, cell death, and interferon gamma and GM-CSF protein secretion, changes that were prevented by knockdown of RNASEL (Fig 5D–5G).

Expression of the IRF transcription factors, especially IRF7, was increased by mutant OAS2. We knocked down IRF7 (Fig 5H) and found a similar prevention of cell death (Fig 5I), indicating that the signaling pathway activated by mutant OAS2 also involves IRF7, a distal member of the viral-detection signaling pathway. Knockdown of IRF3 (Fig 5J), which often acts together with IRF7, had the opposite effect (Fig 5K), suggesting IRF3 acts to oppose signaling via the OAS2 pathway.

## Discussion

These experiments show that the *Oas2* mutation caused activation of OAS2 driven signaling to prevent the activation of lactation in the post partum period. The effect of the mutation could be detected via Stat1 activation from mid pregnancy and was most apparent in the post partum period, and was only required in the mammary epithelial cell for effect. The mutation increased RNase L activity but the enzymatic activity of mutant OAS2 was unaltered. Thus RNase L activation must occur via mechanisms that increase the effect of 2-5A without a change in its production, such as by reducing 2-5A degradation, increasing the efficiency of 2-5A interaction with RNase L or OAS2 interaction with dsRNA, or by causing relief of a mechanism that sequesters 2-5A. The activation of RNAse L is not sufficient to degrade the ribosomes, indicating that the loss of milk production does not occur via a generalized loss of translation. Thus while RNase L expression is required for activity of the mutation, the mutation may act via regulatory mechanisms that do not require 2-5A activation of RNase L. RNase L may be simply permissive of an alternative mechanism of action, such as altering interactions of OAS2 with its cellular binding partners, by changing its subcellular localization, or by decreasing the rate of OAS2 degradation. Thus it is possible that RNase L and OAS2 could also both be involved in as yet undiscovered molecular complexes that initiate activation of this pathway. For example OAS2 has been reported to bind NOD2 [3], and the composition and mechanism of action of this mitochondrial-signaling complex is currently the subject of intense worldwide study, but its definition is proving to be elusive. The non catalytic OAS1b [11] and OASL1 [12] have mechanisms independent of 2-5A production involving molecular interactions. This is the first genetic demonstration that OAS2 can signal in ways other than by alterations in enzyme activity. This mutation may prove to be important for the discovery of the mechanisms signaling the detection of viral infection, which remain largely unknown, because it provides a single point of pathway activation, unlike the existing reagents used for this purpose. Like other family members, OAS2 may regulate apoptosis independently of its function to control viral replication [6,7].

Lactation failure and milk stasis characterize mastitis, raising an interesting new avenue of investigation opened by our findings. The major consequence of mastitis is reduced weight-gain of the infant, precipitating a switch to bottle-feeding where available, or reduced neonatal health where it is not. Our results raise the possibility that the OAS2 pathway may be involved in its pathogenesis. Bacterial infection is commonly thought to be the cause of mastitis but the evidence resoundingly shows that bacterial infection is the sequelae of an unknown primary cause of the disease. For example, in women the severity of symptoms of mastitis do not correlate with the level of bacterial infection, the disease is often observed in the absence of bacteria in the milk, bacteria are often found in the milk of healthy mothers, and meticulous hygiene or prophylactic antibiotics do not prevent mastitis (reviewed [13]). Recent Cochrane Systematic Reviews concluded that there is insufficient evidence to support antibiotic use for the prevention [14] or treatment [15] of mastitis. The strongest risk factors for mastitis in women involve incomplete or interrupted milk flow from one or more galactophores [13] and the World Health Organization recognizes milk stasis as the cause of mastitis [16]. Thus bacterial infection most likely represents progression of mastitis to a more pathogenic form involving abscess formation, but it is not the primary cause.

The concept of physiological inflammation as the primary cause of mastitis was proposed in 2001, though no mechanism was proposed at the time [17], and the unavailability of breast tissue from women with mastitis makes the study of mechanism near impossible. Using mice, Ingman and colleagues hypothesize that molecular pattern receptors like *Tlr4* recognize molecules released by tissue damage caused by milk engorgement, which trigger an innate immune response and milk stasis [13,18]. Alleles of *Tlr4*, a bacterial associated molecular pattern receptor, are linked with the occurrence of mastitis in cattle [19]. *Tlr4* has also been linked to a number of the systemic symptoms of mastitis [13]. As we show, stimulation of the OAS2 pathway can produce the accepted cause of mastitis, milk stasis, opening a new avenue of investigation into human mastitis as a disease amenable to anti-inflammatory therapy. Our findings also open the question of the role of viruses in the initiation of mastitis. Even non-infectious forms could play a role. Fragments of the mouse mammary tumor virus are present in the genome of all laboratory mice and they continue to produce transcripts in response to the hormones of pregnancy, while homologous fragments exist in the human genome [20,21], which may promote milk stasis and inflammation via OAS2 activation.

This is the first time that a viral recognition pathway has been implicated in the regulation of lactation. Transmission of viruses via milk is a well-documented phenomenon and the evolution of mechanisms to prevent it would be expected. This would not necessarily be lethal for the neonate as all mammals have multiple and independent lactation systems. Mice, for example have 10 mammary glands each containing a single ductal system. Each human breast contains between 6 and 8 independent ductal systems, exiting at the nipple without joining. Viral infection in one ductal system, or one mammary gland, could initiate milk stasis in that system, leaving the others to continue lactation. Social systems in humans and mice allow the feeding of neonates by multiple mothers. There could be an intriguing evolutionary twist here resulting from the evolutionary arms-race between viruses and their hosts [22]. Since HIV transmission via the milk occurs far more frequently if mastitis is present [23], could viruses have adapted to this defense and learned to induce a limited mastitis to aid viral transmission?

A molecular mechanism is suggested by our results (S9 Fig) that requires further investigation. STAT1 activation (Fig 1), presumably resulting from the production of interferon due to OAS2 pathway activation, would be expected to cause the induction of the SOCS proteins, which inhibit STAT phosphorylation via targeting the JAK kinases, including JAK2 which phosphorylates STAT5 in response to prolactin, the major hormone driving the onset of lactation. Many aspects of this pathway have been demonstrated in mice such as the regulation of lactation by prolactin via STAT5 [24,25] and the SOCS proteins [26–28], the induction of STAT1 in conditions of sterile mastitis [29] and the ability of STAT1 to regulate prolactin signaling [30]. In the T47D transcript profiling (S6–S8 Figs and S2 Table) we observed increases in the levels of SOCS 1,4,5 and 6. In the ENU mice we observed a decrease in STAT5 phosphorylation. So it is possible that OAS2 pathway stimulation, resulting from the natural rise in OAS2 at d18.5 of pregnancy (S1 and S5 Figs), produces a persistent interferon response in OAS2 mutant animals, because mutant OAS2 activates RNase L which via the resulting interferon response maintains high OAS2 levels, establishing a positive feed-back loop which then persistently prevents prolactin from activating STAT5 (maybe via SOCS) to induce the activation of milk secretion during the post partum period.

## Materials and methods

### Ethics statement

All mice were housed in specific pathogen-free conditions at the Australian Phenomics Facility and the Garvan Institute, with all animal experiments carried out according to guidelines contained within the NSW (Australia) Animal Research Act 1985, the NSW (Australia) Animal Research Regulation 2010 and the Australian code of practice for the care and use of animals for scientific purposes, (8th Edition 2013, National Health and Medical Research Council (Australia)) and approved by either the Australian National University or Garvan/St Vincent’s Animal Ethics and Experimentation Committees (approval number 14/27).

### Mice

ENU mutagenesis and pedigree construction was carried out as previously described [31]. The *Oas2* mutation was discovered in a single G1 female and heritability of the phenotype confirmed by breeding with CBA CaJ male and cross fostering of pups. For quantification of lactation failure litters were standardized to 7 pups per dam. Pups were weighed, as a group, at the same time daily. Mice were injected with BrdU dissolved in H_2_O (100μg BrdU per gram body weight) 2 h prior to sacrifice by CO_2_ asphyxiation, and mammary glands were collected. Mammary glands were either whole-mounted and stained with Carmine alum or snap frozen in liquid nitrogen for mRNA and protein analyses. All animals were housed with food and water ad libitum with a 12-h day/night cycle at 22°C and 80% relative humidity.

### Histopathology and organ pathology

A complete analysis of the histology and pathology of the Jersey strain was conducted by the Australian Phenomics Network (APN) Histopathology and Organ Pathology Service, University of Melbourne. Eight week and a 31 week female sibling pairs, comprised of mt/mt and wt/wt siblings, were examined macro and microscopically. Mammary tissue, ovaries, oviducts, bicornuate uterus, cervix, urinary bladder, liver/gall bladder, cecum, colon, spleen/pancreas, mesenteric lymph node, stomach, duodenum, jejunum, ileum, kidney/adrenal, salivary glands/lymph nodes, thymus, lungs, heart, skin, eyes, brain, spinal cord, skeletal muscle, skeletal tissue/hind leg were macro and microscopically examined.

### Mapping and sequencing

A pool of 15 affected N2 mice and a pool of 15 unaffected N2 backcrossed mice were screened with a set of ~130 markers polymorphic between C57BL/6 and CBA/CaJ mice that spanned the genome at 10–20 Mb intervals. Allele specific SNP primers were designed from a set of validated SNPs available at www.well.ox.ac.uk/mouse/INBREDS/. SNP genotyping was performed using the Amplifluor kit (Chemicon) as per the manufacturers instructions. The confirmation and fine mapping were performed using Amplifluor markers designed to amplify C57BL/6 x CBA/CaJ SNPs within the linkage interval in individual affected and unaffected mice. Markers were designed at approximately 1–2 Mb distances within the initial map interval. More than 250 mice were screened from many successive cohorts of mice from backcrosses to CBA/CaJ to narrow the region to a 3 Mb interval. Sequencing of candidate genes was performed to locate the causal ENU base substitution using an affected mouse homozygous for the linkage region. Primers were designed for candidate genes to amplify all exons +/- 15 bp to cover splice junctions. Sanger sequencing was used to identify the causal mutation by comparing the sequence of the affected individual against a C57BL/6 mouse reference genome. Mutations were confirmed in a second affected individual and a C57BL/6wild type mouse.

### Nimblegen sequence capture

The mapping was performed with the *gsMapper* program, which is part of the 454 software suite. The two samples (defined by Jersey_F4IC140 and Jersey_pool) were mapped against the full region of the mouse genome on chromosome 5. The sequence used as reference is from *genbank build37*/*UCSC mm9*. Further analysis then focused on the reads mapped onto the target region: 118710087–123738720 on chromosome5.

Variation analysis detected where at least 2 reads differ either from the reference sequence or from other reads aligned at a specific location. SNPs, insertion-deletion pairs, multi-homopolymer insertion or deletion regions, and single-base overcalls and under calls are reported. Also, in order for a difference to be identified and reported, there have been at least two non-duplicate reads that (1) show the difference, (2) have at least 5 bases on both sides of the difference, and (3) have few other isolated sequence differences in the read. Variations were classified as high-confidence if they fulfilled the following rules: 1. There must be at least 3 non-duplicate reads with the difference. 2. There must be both forward and reverse reads showing the difference, unless there are at least 5 reads with quality scores over 20 (or 30 if the difference involves a 5-mer or higher). 3. If the difference is a single-base overcall or under call, then the reads with the difference must form the consensus of the sequenced reads (*i*.*e*., at that location, the overall consensus must differ from the reference).

### *Oas2* mutant mouse line maintenance and genotyping

After identification of the causative mutation genotyping was performed using the following primers:

Forward-wildtype: GCTCTTCCTAAAGCAGAT
Forward-mutant: GCTCTTCCTAAAGCAGAA
Common reverse: GGTGTCAGAATTCAAGAAGCAGAC

The *Oas2* mutant colony was maintained by breeding heterozygous males (wt/mt) with wt/wt females. For the generation of homozygous experimental animals wt/mt males were bred with wt/mt females and their offspring removed at 1dpp and fostered on a control mother.

### RNA extractions and quantitative PCR

Total RNA was isolated using Trizol reagent (mouse tissues; Gibco/Invitrogen Vic) or RNeasy extraction kit (cell pellets; Qiagen) according to the manufacturer’s instructions. All total RNA samples were quantified with a Nanodrop 1000 Spectrophotometer (ThermoFisher) prior to loading 100 ng of total RNA on a 2100 Bioanalyzer (Agilent) total RNA analysis. Single stranded cDNA was produced by reverse transcription using 1 μg of RNA in a 20μl reaction and diluted 1:5 with H_2_O (Promega). Quantitative PCR was performed using the Taqman probe-based system (Table 1) on the ABI 7900HT as per the manufacturer’s instructions (Applied Biosystems).

**Table 1:** Taqman probes used for quantitative PCR indicating gene name, probe ID number and species specificity.

Gene	Probe (s)	Species
*Oas2*	Mm00460961_m1	Mouse
*Oas2-2*	Mm01202789_m1	Mouse
*Wap*	Mm00839913_m1	Mouse
*beta Casein*	Mm0089664_m1	Mouse
*RNASEL*	Hs00221692_m1	Human
*IRF7*	Hs01014809_g1	Human
*GapDH*	Hs02758991_g1	Human
*GapDH*	Mm99999915_g1	Mouse

### RNase L dependent tRNA cleavage assay

tRNA cleavage by RNaseL was performed using the technique developed by JD and AK [9]. Briefly, RNA was ligated with 2’,3’-cyclic phosphate to an adaptor with RtcB as described in [9]. Reactions were stopped by adding EDTA and used as a template for reverse transcription with Multiscribe RT. A primer with a 3’-end complimentary to the adaptor and a 5’-overhang that serves as a universal priming site (5’-TCCCTATCAGTGATAGAGAGTTCAGAGTTCTACAGTCCG- 3’) was used for reverse transcription. SYBR green-based qPCR was conducted using a universal reverse primer that binds to the cDNA overhang (underlined) and cleavage-site specific forward primers designed for tRNA [9]. qPCR was carried out for 50 cycles using 62°C annealing/extension for 1 min. U6, which has a naturally occurring 2’,3’-cyclic phosphate and an RNase L independent cleavage site in tRNA-His (position 18, transcript numbering; [9]) was used for normalization.

### Cell free *Oas2* poly (I:C) sensitivity assay

HeLa cells were maintained in MEM + 10% FBS in a humidified 5% CO_2_ atmosphere. Cells cultured in 10 cm dishes were transfected at 80–90% confluence with 10 μg empty pcDNA4/TO or N-terminally FLAG-tagged WT or I405N mouse OAS2 in pcDNA4/TO (Life Technologies) using Lipofectamine 2000 (Life Technologies). Cells were harvested by trypsinization 24h post-transfection, resuspended in complete media, and washed 2 x 10 mL ice-cold PBS. Cell pellets were lysed in buffer A (20 mM HEPES pH 7.5, 100 mM NaCl, 0.1% Triton X100, and 1x complete protease inhibitor cocktail (Roche) for 10 min with end-over-end rotation at 4°C. Lysates were cleared by centrifugation at 16,000 x g, 15 min, 4°C and the supernatants subjected to immunoprecipitation with M2-α-FLAG magnetic beads (Sigma) for 2h at 4°C followed by 4 x 1mL washes, 10 min per wash, with buffer A without protease inhibitors. After the fourth wash the beads were washed with 2 x 1 mL storage buffer (20 mM HEPES pH 7.5, 100 mM NaCl, 10% glycerol), the supernatant removed, storage buffer added to 100 μL. Inputs and IPs were blotted with α-FLAG to verify expression and IP of FLAG-OAS2. 5% of each IP was incubated with 1 mM ATP, trace-labeled with 3 nM ^32^P-α-ATP, in the absence or presence poly (I:C) (Sigma) for 2h at 37°C. Reaction volumes were 20 μL and contained 20 mM HEPES pH 7.5, 70 mM NaCl, 10 mM MgCl_2_, 10% glycerol, and 4 mM DTT. After incubation, the reactions were quenched by adding 120 μL stop buffer (8M urea, 0.1% SDS, 1 mM EDTA, 0.02% bromophenol blue, 0.02% xylene cyanol). Equal portions of each reaction were resolved by 20% denaturing PAGE and visualized by phosphoimaging. Gels were quantitated using GelQuant.NET software.

### Mouse *Oas2* cloning and cell culture experiments in T47D human breast cancer cell lines

The N—terminal and C—terminal cDNAs of mouse *Oas2* were obtained as a gift of Yoichiro Iwakura (Institute of Medical Science, University Tokyo) and subcloned into pcDNA3.1 and pBluescript vectors. Site directed mutagenesis was performed using Phusion Site-directed mutagenesis (Thermo Scientific) as per the manufacturer’s instructions using the following primers (Forward Jer: TATATGTTCCTTCCTTAAAAATGTCTGC and Reverse AGGATTTCGTCTTGTTCCTTCGACAACTGTA). Wildtype and mutant (I405N) mouse *Oas2* clones were then subcloned into pShuttle and finally into the pHUSH ProEx tetracycline inducible retroviral expression system [32]. Retrovirus was then packaged by transfecting Phoenix cells with pHUSH containing either mouse wildtype (wt) or *Oas2* I405N mutant (mt) cDNAs using Fugene transfection reagent (Promega). T47D breast cancer cells were then infected with filtered viral supernatants and stable cell lines selected using Puromycin. T47Ds were maintained sub-confluent in RPMI complete media (Gibco) containing 10% tetracycline free FCS and supplemented with 10μg/ml Insulin. Mouse *Oas2* wt or mt expression was induced with 100 ng/ml Doxycline (DOX) or vehicle control daily in the media and cells were harvested at 72 hours after plating. Cell counts of viable and non-viable cells (identified by the incorporation of 0.4% Trypan Blue at a 1:2 dilution) were performed in triplicate from 3 independent experiments. Annexin V PI staining was performed using the Annexin V-FITC Apoptosis Kit (Biovision, CA USA) as per the manufacturers instructions. Human inflammatory cytokines were analyzed using the Multi-Analyte ELISArray (Qiagen).

### HC11 Mouse *Oas2* cloning and cell culture experiments

Retrovirus was made packaged as above by transfecting Phoenix cells with pHUSH containing either mouse wildtype (wt) or Oas2 I405N mutant (mt) cDNAs using Fugene transfection reagent (Promega). HC11 normal mouse mammary cell lines were then infected with filtered viral supernatants and stable constitutive cell lines selected using Puromycin and then clonal colonies established by titrating single cells into 96 well plates. HC11s were maintained in maintenance RPMI media containing 10% FCS and supplemented with 5μg/ml Insulin and 10ng/ml human recombinant epidermal growth factor (EGF, Sigma-Aldrich). For differentiation assays, HC11 cells were plated sub confluent in maintenance media for 2 days until confluent and then media replaced with RPMI media containing 10% FCS and supplemented with 5μg/ml Insulin and supplemented with 5μg/ml sheep pituitary Prolactin (Sigma Aldrich) and 1μM Dexamethasone (Sigma Aldrich) daily for 3 days before RNA harvest and quantitative PCR analysis as above. For transient transfections apoptotic assays wildtype (wt) or Oas2 I405N mutant (mt) cDNAs were cloned into pIRES-EGFP vectors and transiently transfected with 2μg DNA/well using X-tremeGENE transfection reagent (Roche) in maintenance media as per the manufacturers instructions. After 24 hours the media replaced with RPMI media containing 10% FCS and supplemented with 5μg/ml Insulin and cells were harvested 96 hours after transfection for Annexin V PI staining as above.

### Poly (I:C) sensitivity assay

T47D *Oas2* wt or mt cells were plated in 10cm dishes and treated daily for 48 hours with 100 ng/ml DOX or vehicle. At 48 hours the cells harvested by trypsinization, retreated with DOX or vehicle, counted, plated at a density of 5000 cells/well and simultaneously transfected with Poly (I:C) (11 point titration) using RNAiMAX (Invitrogen) in opaque 96 well plates. 24 hours after transfection the plates were analysed using the CellTiter-Glo Luminescent Cell Viability Assay Protocol (Promega). Inhibitory dose curves were plotted in Prism 6 statistical software and normalized data analyzed using the sigmoidal-dose response function. The mean was calculated from quadruplicate replicates.

### Replating assay

T47D *Oas2* wt or mt cells were plated in T150 flasks and mouse *Oas2* wt or mt expression was induced with 100 ng/ml DOX or vehicle for 48 hours prior to trypsinisation by 0.25% Trypsin (no EDTA) with phenol red (Life Technologies) for 3 mins. 1 x 10^6^ cells from each cell line was treated with DOX or vehicle and then plated in 6-well plates and allowed to adhere for 4 hours. After 4 hours the cells were gently washed, trypsinised and the number of adherent cells counted and expressed as a proportion of the total number of cells plated.

### Propidium iodide cell cycle analysis

T47D *Oas2* wt or mt cells were plated in 6-well plates and mouse *Oas2* wt or mt expression was induced with 100 ng/ml DOX or vehicle for 72 hours. Cells were harvested by trypsinisation, washed in 1 ml of PBS and fixed by adding 10 mls of 100% cold ethanol drop wise onto 1ml re-suspended cells and incubated at 4°C overnight. Cells were then pelleted, washed and incubated at 90°C for 5 mins and then re-suspended in a FACS buffer containing 0.5ng/ml RNase (Qiagen) and 1μg/ml Propidium iodide. Flow cytometry was performed and G1, S phase and G2/M phases for each experimental group estimated using the propidium iodide fluorescence intensity histograms. The mean of 5 independent experiments was calculated.

### siRNA experiments

T47D *Oas2* wt or mt cells were reverse transfected with ON-TARGET plus SMARTPOOLS of siRNA against RNaseL (L-005032-01-05) or Non Targeting controls (D-001810-10-05) using RNAiMAX (Invitrogen) in 10cm dishes as per the manufacturers specification. 24 hours after transfection, cells were washed, media replaced and either treated with 100 ng/ml DOX or vehicle daily for 3 days after which they were harvested by trypsinisation. Annexin V PI staining was performed using the Annexin V-FITC Apoptosis Kit (Biovision, CA USA) as per the manufacturers instructions. Cell pellets were also collected for RNA isolation and western blotting and the supernatant collected, filtered with a 0.22μm filter and stored at -80°C. Human inflammatory cytokines were analyzed using the Multi-Analyte ELISArray™ Kits as per the manufacturers instructions.

### Transcript profiling

Wt/wt or mt/mt mice were time mated and mammary glands collected at day 18 of pregnancy or 2 days after partuition (2dpp) and snap frozen in liquid N_2_. Total RNA was isolated using Trizol reagent (Gibco/Invitrogen, Vic) and measured on the 2100 Bioanalyzer (Agilent). From the cell experiments, total RNA was extracted using the RNeasy extraction kit (Qiagen) for cells with or without DOX induction of the wt or mt *mOas2* gene. Total RNA from the mouse mammary glands was then labeled and hybridized to the Mouse Transcriptome Array (MTA) 1.0 as per the manufacturer’s instructions (Affymetrix Ca, USA) at Ramiaciotti Centre for Genomics (UNSW Sydney, Australia). Likewise, total RNA from the T47D cells was labeled and hybridized to the Affymetrix Human Transcriptome Array (HTA) 2.0 as per the manufacturer’s instructions (Affymetrix Ca, USA) at Ramiaciotti Centre for Genomics (UNSW Sydney, Australia). All mouse and T47D samples were prepared in biological triplicate for each experimental grouping, except for the T47D mt -DOX group where analysis was performed in duplicate due to one of the samples failing quality control. Microarray data are freely available from GEO: GSE69397 http://www.ncbi.nlm.nih.gov/geo/query/acc.cgi?token=ohkleoespjotzoh&acc=GSE69397

Quality control was performed using the Affymetrix Expression Console. Normalisation and probe-set summarization was performed using the robust multichip average method of the Affymetrix Power Tools *apt-probeset-summarize* software (version 1.16.1) (using the -a rma option). The transcript clusters with official HGNC symbols were then extracted from the HTA 2.0 arrays, resulting in 23532 gene transcript clusters. Differential expression between experimental groups was assessed using Limma [33] via the limmaGP tool in GenePattern. Functionally associated gene-sets were identified using Gene Set Enrichment Analysis (GSEA) [34] on a ranked list of the limma moderated t-statistics, from each pair-wise comparison, against a combined set of 6947 gene-sets from v4.0 of the MSigDB [35] and custom gene-sets derived from the literature. Mouse gene-symbols were mapped to their human orthologs using the ensembl database. The Enrichment Map plugin [36] for Cytoscape [37] was used to build and visualize the resulting regulatory network of gene-signatures, with conservative parameters: p = 0.001; q = 0.05; overlap s = 0.5.

### Polyacrylamide gel electrophoresis and Western blotting

10μg reduced protein was loaded in each well of 12% NuPAGE SDS polyacrylamide gels (Life Technologies) and separated using electrophoresis. Proteins were transferred to Immun Blot PVDF (Biorad) and Western blotted for mouse *Oas2* (M-105, sc99098 Santa-Cruz), RNaseL (H-300, sc25798 Santa Cruz), E-cadherin (610182 BD Biosciences) and beta-ACTIN (AC-74, A5316, Santa Cruz)

### Cluster generation using self-organising maps

The limma F-test statistic [33], with a Benjamini-Hochberg adjusted p-value threshold of 0.05, was used to identify differentially expressed transcripts across the four experimental groups in the mouse expression arrays (wt/wt 2dpc, wt/wt 2dpp, mt/mt 2dpc, mt/mt 2dpp) and T47D cell-line expression arrays (wt–Dox, wt +Dox, mt–Dox, mt +Dox). This resulted in 660 and 135 significant transcript clusters from the mouse and T47D arrays, respectively.

Self-organising maps (SOMs), consisting of 6 nodes, were used to identify clusters of genes in both the mouse and T47D cells. The z-scores of the log2 normalised gene-expression values, for each transcript cluster, were used as input to the biopython SOM algorithm implementation [38]. The *somcluster()* parameters used were: iterations = 50,000; nx = 2, ny = 3, inittau = 0.02, dist = Euclidean.

### DAVID functional annotation clustering

The *db2db()* function from the BioDBNet database [39] was used to convert gene-symbols to Ensembl gene IDs for input into DAVID. Functional annotation clustering was carried out using the *getTermClusterReport()* function from the DAVID web services interface (Jiao et al., 2012), with the following parameters: overlap = 3, initialSeed = 3, finalSeed = 3, linkage = 0.5, kappa = 50.

DAVID databases used: (BBID, GOTERM_CC_FAT, BIOCARTA, GOTERM_MF_FAT, SMART, COG_ONTOLOGY, SP_PIR_KEYWORDS, KEGG_PATHWAY, INTERPRO, UP_SEQ_FEATURE, OMIM_DISEASE, GOTERM_BP_FAT, PIR_SUPERFAMILY)

### Functional enrichment of signature gene-sets

A Hypergeometric test was used to calculate the level of gene overlap between the genes identified in each SOM cluster and the MSigDB gene-set collections [35] and our custom functional signature gene-sets. A background set number, of 45956, as described on the MSigDB website, was used. A Benjamini-Hochberg (BH) corrected p-value was calculated for each set and a threshold of BH<0.05 was considered a significant enrichment. Mouse gene-symbols were mapped to their human orthologs using the ensembl database.

### Mammary gland whole mounting and immuno-histochemistry

Mouse mammary glands were harvested from wt/wt and mt/mt mice and fixed in 4% buffered formalin for 4 hours. Glands were defatted in 3–4 changes of acetone before being dehydrated and stained in Carmine alum as previously described [40]. Glands were then dehydrated in a series of graded alcohols and embedded in Paraffin for sectioning. Sections were either stained with haematoxylin and eosin for routine histochemistry or stained with antibodies to the following antigens using immunohistochemistry protocols as detailed in Table 2.

**Table 2:** Antibodies, concentration and antigen retrieval conditions for immunohistochemistry. All reagents were from Dako unless otherwise specified. Visualisation of antigen: antibody complexes was performed using the DAB+ liquid Substrate chromogen system (K3467).

Antigen	Antibody	Species reactivity	Retrieval	Primary antibody conc.	Secondary antibody
Oas2	M18, sc49858 Santa-Cruz	Mouse/ human	pH9 S2367 Pressure Cooker 15secs	1:200	Goat Immpress-HRP (Vector Labs) MP7405
Anti Milk	Accurate Chemical & Scientific CO. YNRMTM	mouse	pH6 S1699 Waterbath 20 mins	1:12000	Envision Rabbit (K4009)
Cleaved Caspase 3	Asp175 5A Cell Signaling 9661	Mouse/ human	pH9 S2367 Pressure Cooker 30secs	1:100	Envision Rabbit (K4009)
BrdU	Bu20a M0744 Dako	mouse	pH9 S2367 Waterbath 20 mins	1:100	Envision Mouse (K4007)
P-Stat1	Tyr701 (58D6) Cell Signalling 9167	mouse	pH9 S2367 Pressure Cooker 30secs	1:800	Signal Stain Boost Cell Signalling 8114
P-Stat5	Tyr694 (C11C5) Cell Signalling 9359	mouse	pH9 S2367 Pressure Cooker 30secs	1:600	Signal Stain Boost Cell Signalling 8114

## Supporting information

S1 Fig Mammary phenotype at key stages of mammary development. **(A)** Whole mount histology of the 4^th^ inguinal mammary gland showing ductal development in mature virgin mice (8–10 weeks old) and lobulo-alveolar development at 12.5 days post coitus (dpc), 18.5 dpc and 2 days post partum (2dpp) in wild type mice (wt/wt) or homozygous mutant mice (mt/mt). **(B)** Corresponding hematoxylin-eosin histochemistry. **(C)** Corresponding immunohistochemistry for milk protein expression using an antibodies raised against whole mouse milk. **(D)** Corresponding western blot for milk proteins using the anti mouse milk antibody and keratin 18 loading control. Molecular size is shown together with the established sizes of the indicated milk proteins [41]. Lactoferrin (LF), serum albumin (SA), caseins α,κ,β,γ and ε whey acidic protein (wap) and alpha lactalbumin (αLac). **(E)** Corresponding *Oas2* expression by quantitative PCR for regions of exon 4 (ex4) or exon 10 (ex10) with error bars showing standard error and p values for comparison of wt/wt and corresponding mt/mt animals at the indicated time points.
(TIF)

S2 Fig Global patterns of gene expression in the mammary glands of wildtype and mutant mice. Whole mouse mammary glands from homozygouns *Oas2* mutant (mt) or wild type (wt) animals were interrogated using Affymetrix MTA arrays. Differential gene expression was ranked by LIMMA and this was used as the input for gene set enrichment analysis to identify functional signatures. The enrichment-map plug in for cytoscape was used to visualize the results. Each node represents a gene set and the expression of genes comprising the leading edge of some of these sets is shown as heat maps. Labels indicate the function of the clustered gene sets. **(A)** comparison of gene expression at day 2 post partum (2dpp) with day 18 post coitus (18dpc) in either mutant (mt) mice, color at the node center, or wild type mice (wt), color at the node border. Red indicates enrichment of expression the gene set and blue suppression of expression. **(B**) Comparison of gene expression in mt animals with wt animals at 2dpp, color at the node center, or 18dpc, color at the node border.
(TIF)

S3 Fig Self organizing maps to identify functional expression signatures. **Top panel,** gene expression changes induced in mouse mammary gland by expression of mutant or wild-type *Oas2*, resolved into 6 patterns. **Bottom panel**, corresponding functional groups uniquely contained within each of the gene expression patterns from the top panel. The top-5 functions in each category (DAVID, MolSig DB Hallmark sets, Transcription factor sets (TFT), our set of Involution and lactation profiles and MolSig DB pathways sets) are shown as scored either by the DAVID enrichment score or the BH corrected p-value from the hypergeometric test.
(TIF)

S4 Fig The *Oas2* mutation. **(A**) Details of the mutation in *Oas2* showing amino acid change. **(B**) Conservation of the region containing the mutation in very diverse species including Archaea. **(C)** Location of the mutation in relation to the active enzyme site of Oas2.
(TIF)

S5 Fig Patterns of *Oas2* expression and RNaseL activity during development in mouse mammary gland. **(A**) *Oas2* and *RNaseL* expression in the mammary glands of wild type mice at the indicated stages of mammary development measured by quantitative PCR. **(B)** Immunohistochemistry for OAS2 in wild type mouse mammary glands. **(C)** Bioanalyser results of RNA banding pattern from the mammary glands of individual mice of the indicated genotypes and stages of pregnancy and lactation. RNase L- mediated ribosomal RNA cleavage is compared to non RNase L cleavage using a PCR based method described [9].
(TIF)

S6 Fig Transcript profiling of T47D cells with and without induction of mutant or wild type mouse *Oas2*. **(A**) Comparison of global gene expression patterns between T47D cell lines expressing either mutant (mt) or wild type (wt) forms of mouse *Oas2* when treated with Doxycycline (+DOX) (node center color) or with vehicle (-DOX) (node ring color) for 48 hours. Central map shows network diagram of gene sets with enrichment values indicated by color scale from red to blue as indicated. Heat maps show expression (t-statistic) of the listed genes from the indicated gene sets without (-) or with (+) DOX between mt and wt cells using pink to green color scale as indicated. Functional role of the clusters of gene sets are shown. (**B**) Alternative view of the data showing the comparison +DOX with–DOX within either the mt or wt expressing cell lines. Details as above.
(TIF)

S7 Fig Self organizing maps to identify functional expression signatures. **(Top panel)** Gene expression changes induced in T47D cells by expression of mt or wt *Oas2* resolved into 6 patterns. (**Bottom panel**) Corresponding functional groups uniquely contained within each of the gene expression patterns indicated in the top panel. The top-5 functions in each category are shown as scored either by the DAVID enrichment score or the BH corrected p-value from the hypergeometric test.
(TIF)

S8 Fig Common patterns of changed gene expression induced by mutant *Oas2* in mouse mammary gland and T47D human breast cancer cells. (A) Comparison of global gene expression patterns between mouse 2dpp mammary gland expressing either mutant (mt) or wild type (wt) forms of mouse *Oas2* (node center color) and T47D cell lines expressing either mutant (mt) or wild type (wt) forms of mouse *Oas2* when treated with Doxycycline (+DOX) (node ring color) for 72 hours. Central map shows network diagram of gene-sets with enrichment values indicated by color scale from red to blue as indicated. Functional role of the clusters of gene sets are shown. Outer panels show box-plots of the GSEA normalised enrichment scores (NES) for the each of the gene-sets in the specified functional clusters. Red crosses indicate positively-enriched gene-sets with FDR<0.05, blue crosses indicate negatively-enriched gene-sets with FDR<0.05 and grey crosses indicate gene-sets that have FDR> = 0.05. **(B)** Functional groups found common to mouse and human by self organizing maps.
(TIF)

S9 Fig Proposed mechanism linking OAS2 to the regulation of lactation. **(A)** Heatmap extracted from the T47D transcript profiling showing expression changes in SOCS protein gene expression that exceed F value greater than 0.05. **(B**) Example of immunohistochemistry for STAT5 phosphorylation, which is quantitated in the **(C)** chart for %positivity. Note this quantification does not capture the increased signal intensity observed in wt glands compared to mt glands. **(D)**Diagram at the RHS shows the proposed pathway links between interferon and prolactin signaling that may explain the ability of the mutation in OAS2 to prevent lactation.
(TIF)

S1 Table Table listing the top 5000 probe sets with the greatest differential expression between wt/wt and mt/mt mammary glands collected from mice at 18dpc and 2dpp, detected using the Affymetrix Mouse Transcriptome Assay (MTA) 1.0 GeneChips. Columns provide the MTA transcript cluster ID, change in gene expression between specified time points and genotypes, average expression of each probe across arrays, Limma t-test p-value and adjusted p-value and gene and title symbol where available.
(TXT)

S2 Table Table listing the top 5000 probe sets with the greatest differential expression in T47D cells expressing either wildtype or mutant *OAS2* and induced with DOX and or vehicle control in the presence of non-targeting or RNaseL siRNAs, detected using the Affymetrix Human Transcriptome Assay (HTA) 2.0 GeneChips. Columns provide the HTA transcript cluster ID, change in gene expression between specified treatments, average expression of each probe across arrays, Limma t-test p-value and adjusted p-value, F statistic and gene and title symbol where available.
(TXT)
