# Supplementary figures and images for: A mutation in the viral sensor 2’-5’-oligoadenylate synthetase 2 causes failure of lactation
Source: PLoS Genet. 2017 Nov 8;13(11):e1007072. doi: 10.1371/journal.pgen.1007072 (PMC5695588; doi:10.1371/journal.pgen.1007072)

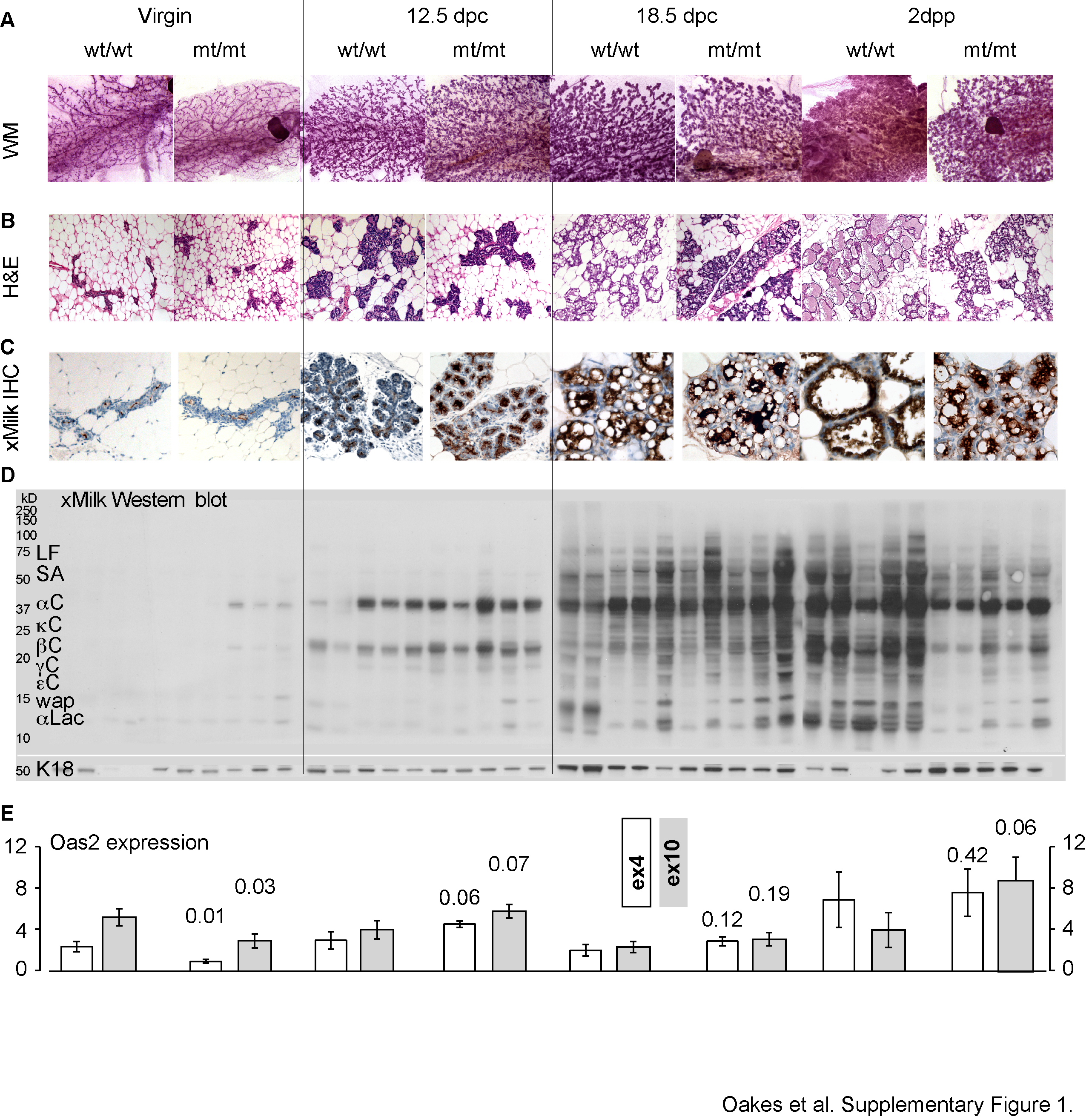

Supplement: S1 Fig — (A) Whole mount histology of the 4th inguinal mammary gland showing ductal development in mature virgin mice (8–10 weeks old) and lobulo-alveolar development at 12.5 days post coitus (dpc), 18.5 dpc and 2 days post partum (2dpp) in wild type mice (wt/wt) or homozygous mutant mice (mt/mt). (B) Corresponding hematoxylin-eosin histochemistry. (C) Corresponding immunohistochemistry for milk protein expression using an antibodies raised against whole mouse milk. (D) Corresponding western blot for milk proteins using the anti mouse milk antibody and keratin 18 loading control. Molecular size is shown together with the established sizes of the indicated milk proteins [41]. Lactoferrin (LF), serum albumin (SA), caseins α,κ,β,γ and ε whey acidic protein (wap) and alpha lactalbumin (αLac). (E) Corresponding Oas2 expression by quantitative PCR for regions of exon 4 (ex4) or exon 10 (ex10) with error bars showing standard error and p values for comparison of wt/wt and corresponding mt/mt animals at the indicated time points. (TIF) [file pgen.1007072.s001.tif]

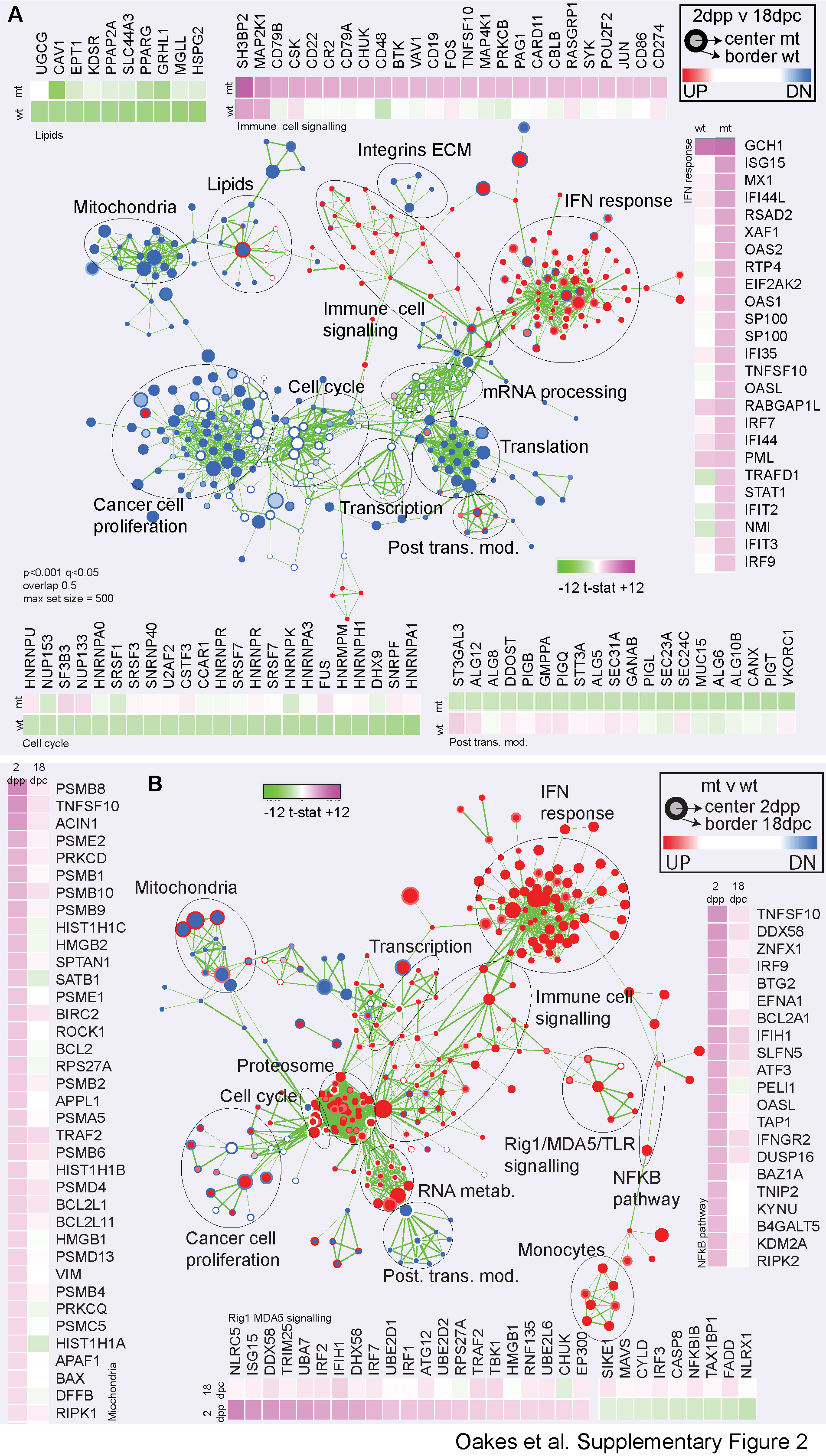

Supplement: S2 Fig — Whole mouse mammary glands from homozygouns Oas2 mutant (mt) or wild type (wt) animals were interrogated using Affymetrix MTA arrays. Differential gene expression was ranked by LIMMA and this was used as the input for gene set enrichment analysis to identify functional signatures. The enrichment-map plug in for cytoscape was used to visualize the results. Each node represents a gene set and the expression of genes comprising the leading edge of some of these sets is shown as heat maps. Labels indicate the function of the clustered gene sets. (A) comparison of gene expression at day 2 post partum (2dpp) with day 18 post coitus (18dpc) in either mutant (mt) mice, color at the node center, or wild type mice (wt), color at the node border. Red indicates enrichment of expression the gene set and blue suppression of expression. (B) Comparison of gene expression in mt animals with wt animals at 2dpp, color at the node center, or 18dpc, color at the node border. (TIF) [file pgen.1007072.s002.tif]

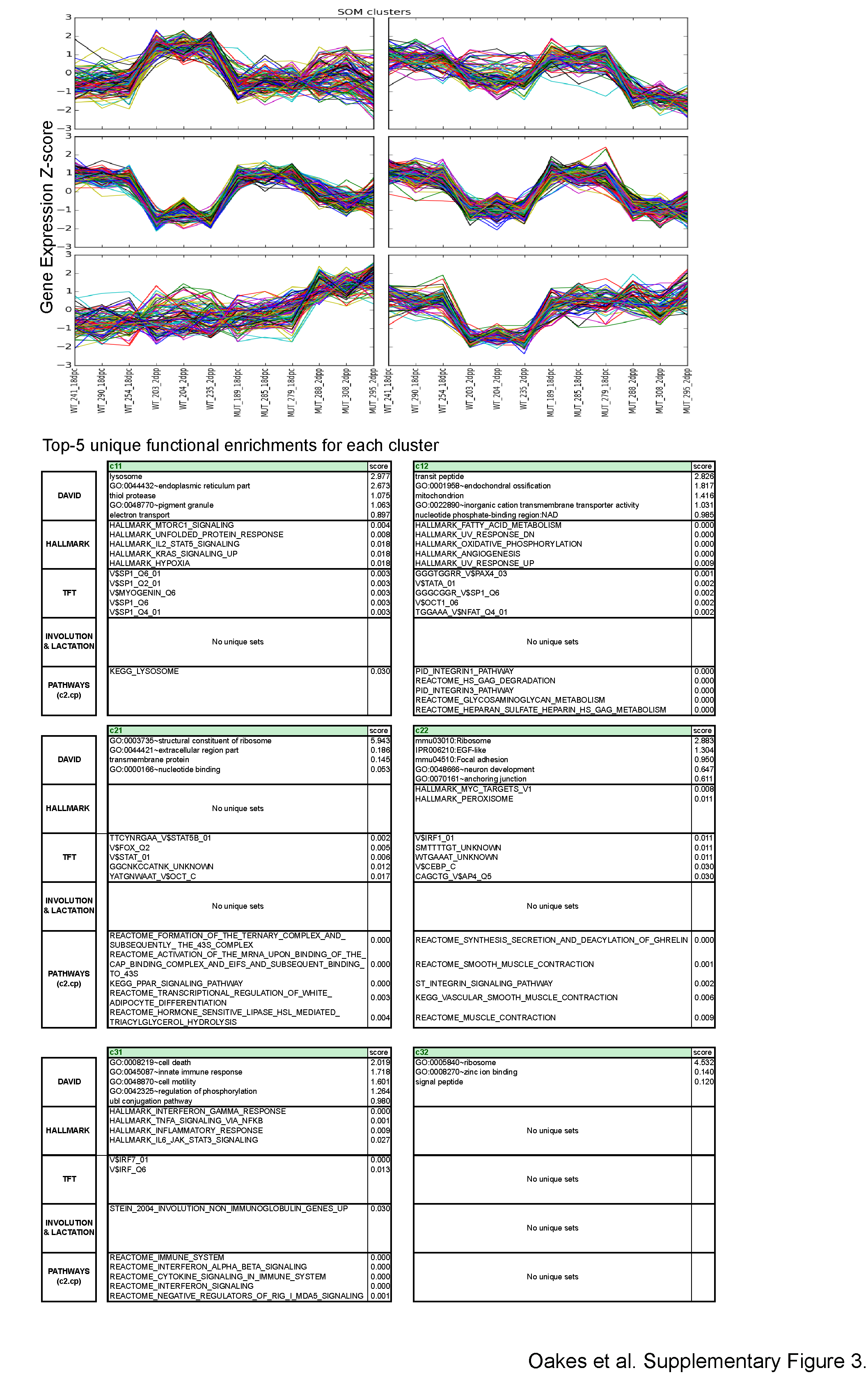

Supplement: S3 Fig — Top panel, gene expression changes induced in mouse mammary gland by expression of mutant or wild-type Oas2, resolved into 6 patterns. Bottom panel, corresponding functional groups uniquely contained within each of the gene expression patterns from the top panel. The top-5 functions in each category (DAVID, MolSig DB Hallmark sets, Transcription factor sets (TFT), our set of Involution and lactation profiles and MolSig DB pathways sets) are shown as scored either by the DAVID enrichment score or the BH corrected p-value from the hypergeometric test. (TIF) [file pgen.1007072.s003.tif]

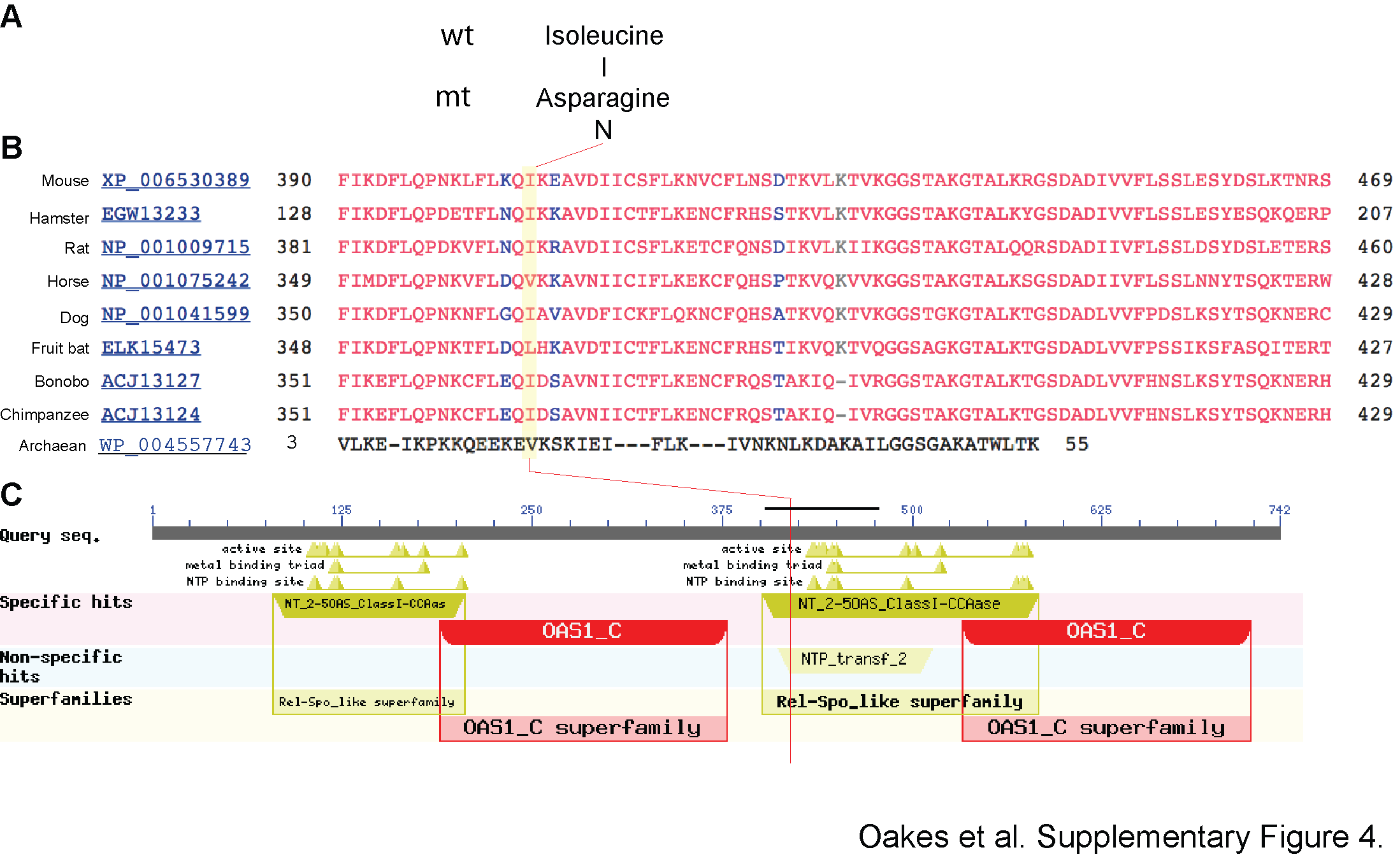

Supplement: S4 Fig — (A) Details of the mutation in Oas2 showing amino acid change. (B) Conservation of the region containing the mutation in very diverse species including Archaea. (C) Location of the mutation in relation to the active enzyme site of Oas2. (TIF) [file pgen.1007072.s004.tif]

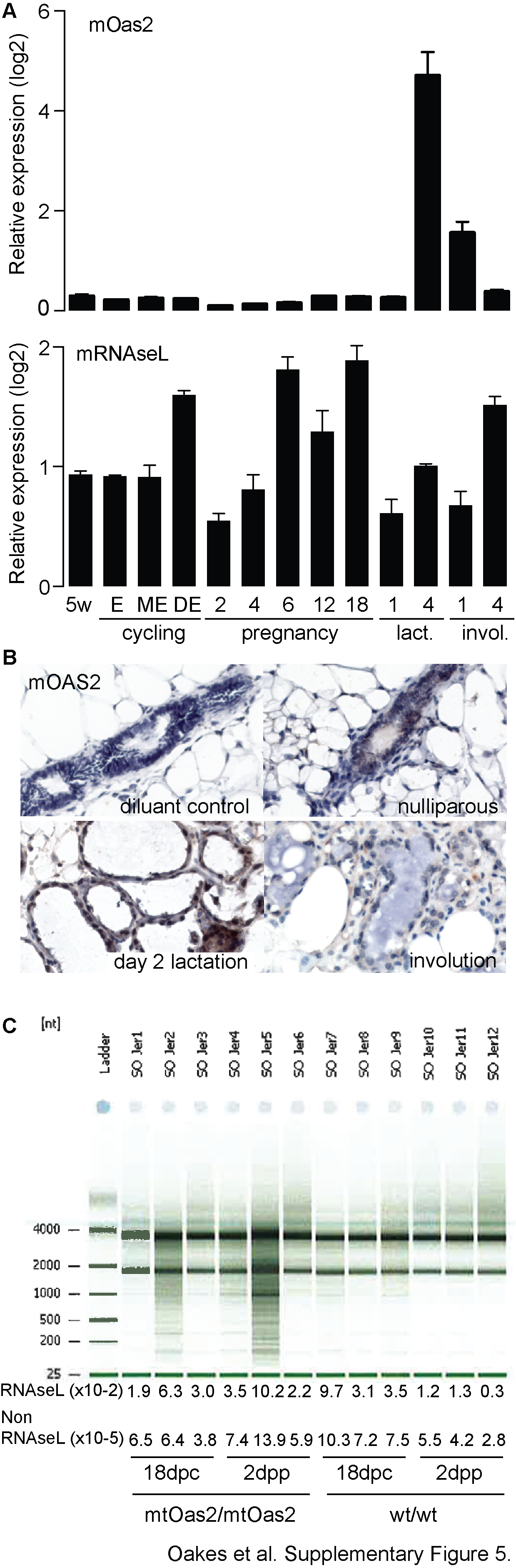

Supplement: S5 Fig — (A) Oas2 and RNaseL expression in the mammary glands of wild type mice at the indicated stages of mammary development measured by quantitative PCR. (B) Immunohistochemistry for OAS2 in wild type mouse mammary glands. (C) Bioanalyser results of RNA banding pattern from the mammary glands of individual mice of the indicated genotypes and stages of pregnancy and lactation. RNase L- mediated ribosomal RNA cleavage is compared to non RNase L cleavage using a PCR based method described [9]. (TIF) [file pgen.1007072.s005.tif]

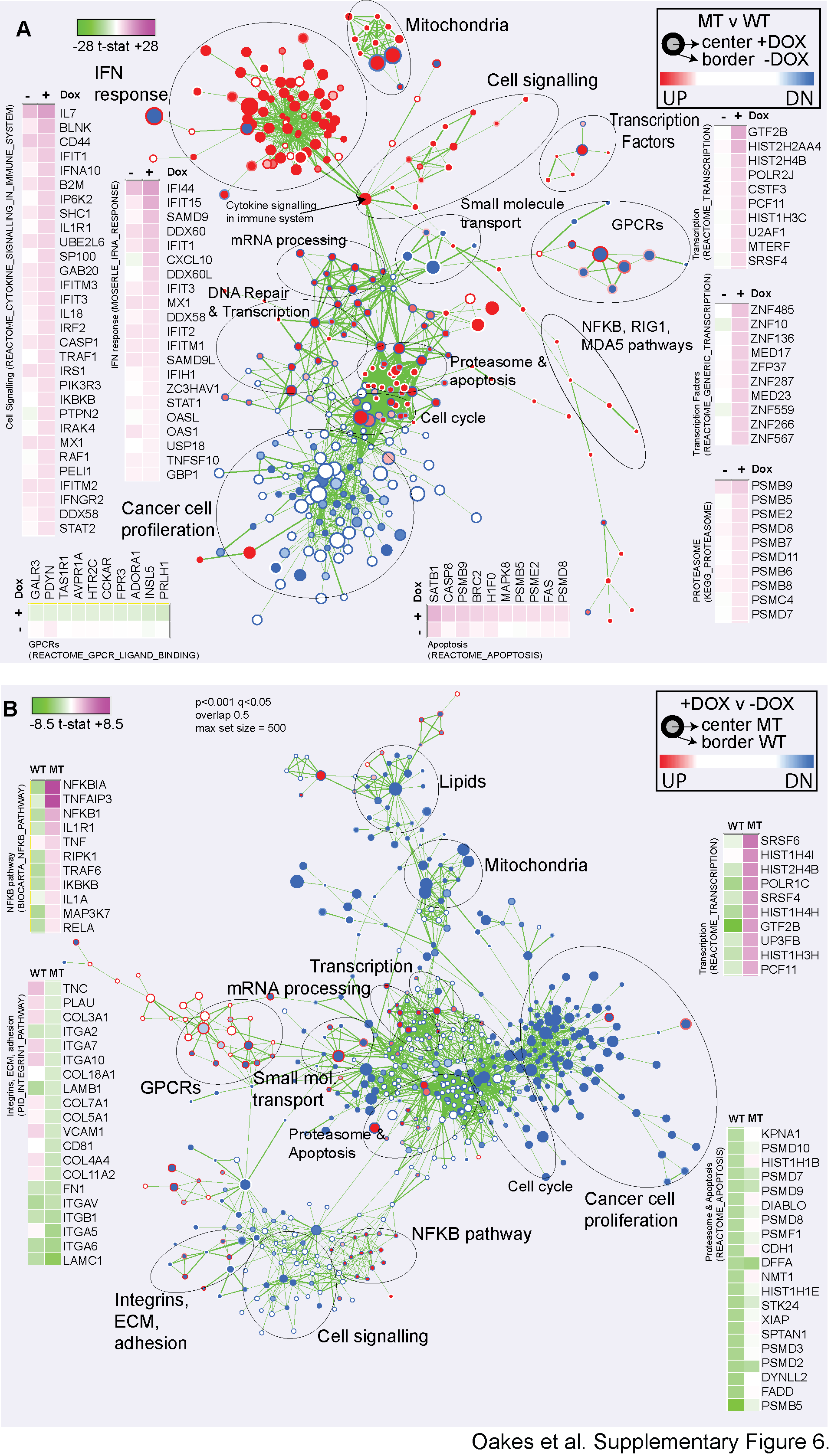

Supplement: S6 Fig — (A) Comparison of global gene expression patterns between T47D cell lines expressing either mutant (mt) or wild type (wt) forms of mouse Oas2 when treated with Doxycycline (+DOX) (node center color) or with vehicle (-DOX) (node ring color) for 48 hours. Central map shows network diagram of gene sets with enrichment values indicated by color scale from red to blue as indicated. Heat maps show expression (t-statistic) of the listed genes from the indicated gene sets without (-) or with (+) DOX between mt and wt cells using pink to green color scale as indicated. Functional role of the clusters of gene sets are shown. (B) Alternative view of the data showing the comparison +DOX with–DOX within either the mt or wt expressing cell lines. Details as above. (TIF) [file pgen.1007072.s006.tif]

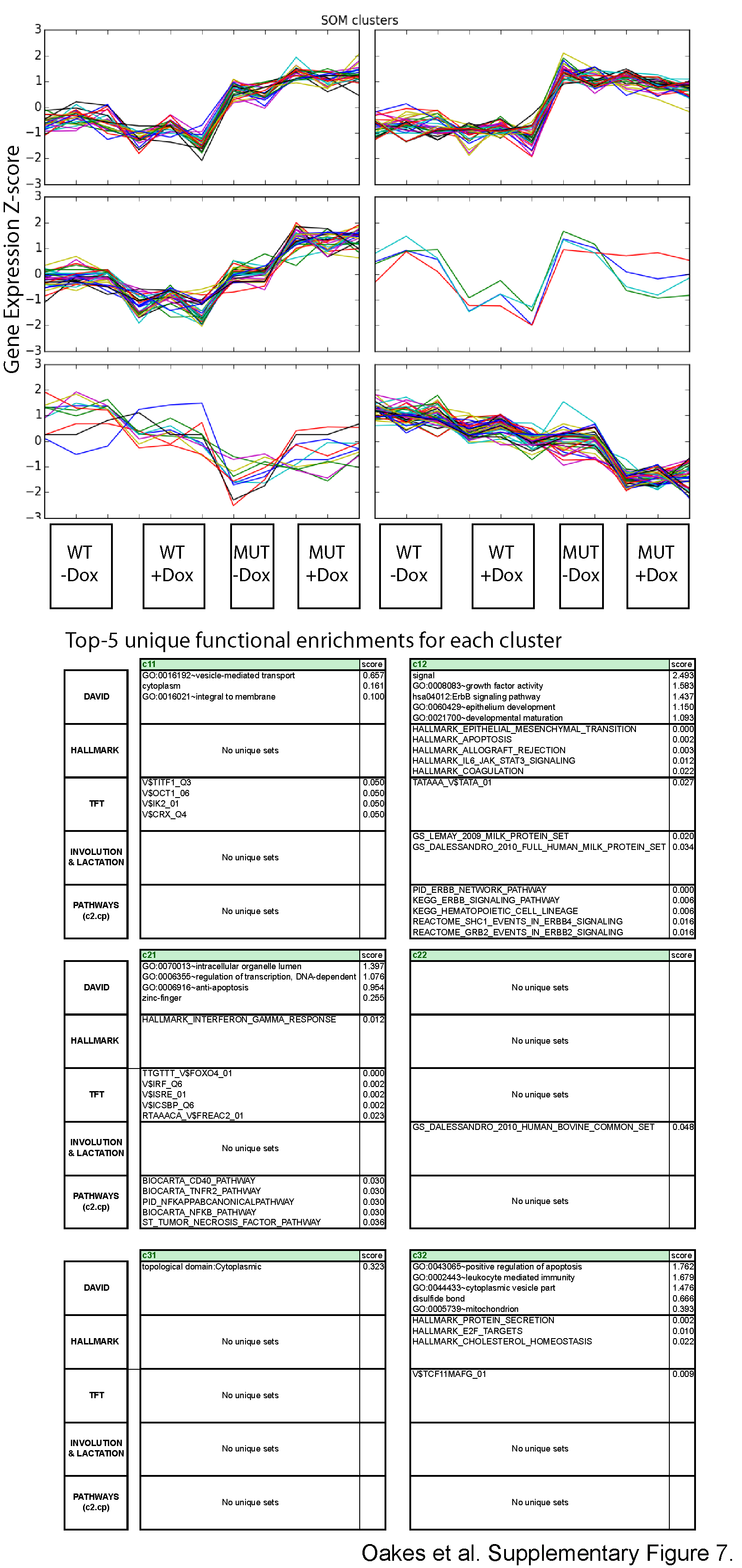

Supplement: S7 Fig — (Top panel) Gene expression changes induced in T47D cells by expression of mt or wt Oas2 resolved into 6 patterns. (Bottom panel) Corresponding functional groups uniquely contained within each of the gene expression patterns indicated in the top panel. The top-5 functions in each category are shown as scored either by the DAVID enrichment score or the BH corrected p-value from the hypergeometric test. (TIF) [file pgen.1007072.s007.tif]

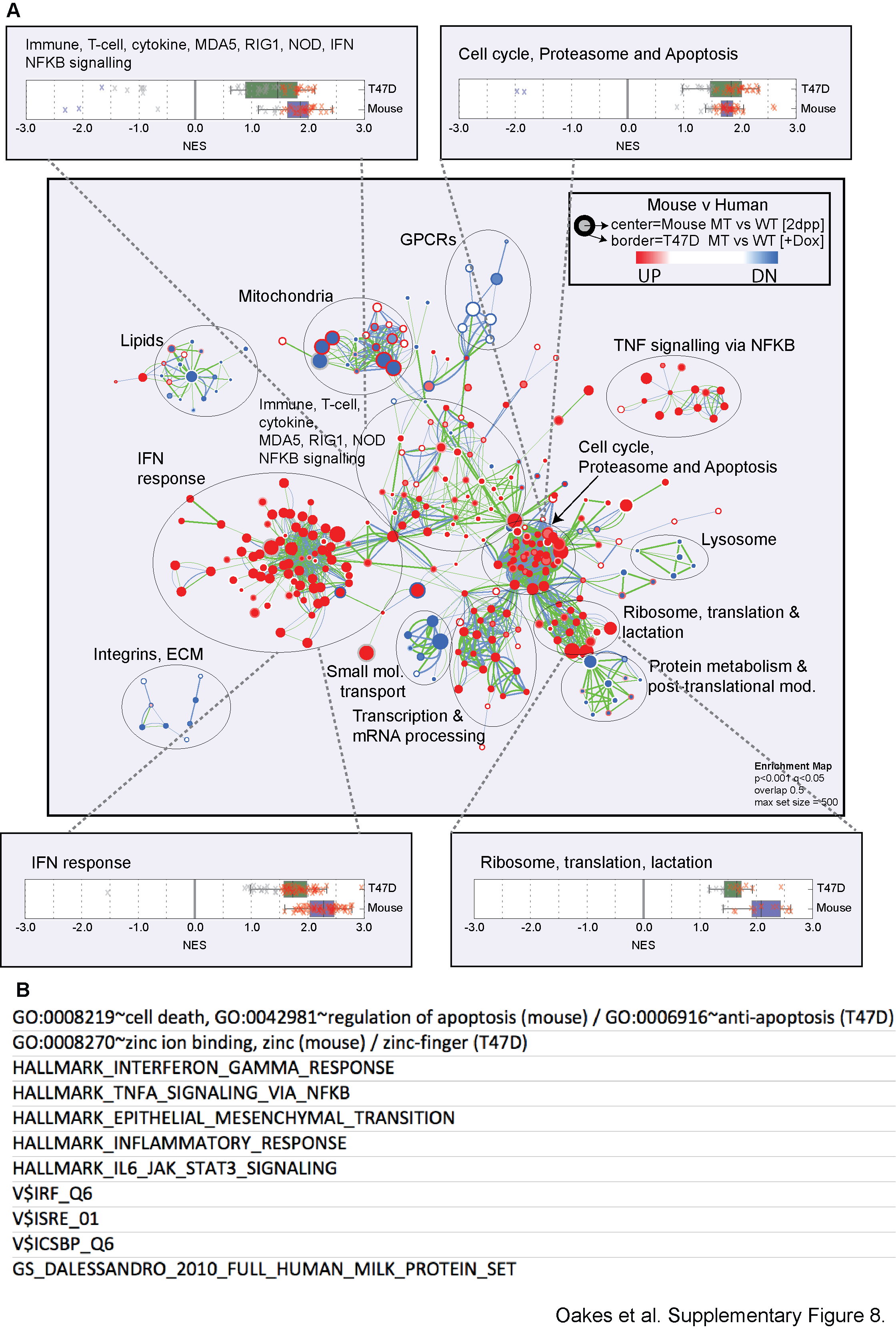

Supplement: S8 Fig — (A) Comparison of global gene expression patterns between mouse 2dpp mammary gland expressing either mutant (mt) or wild type (wt) forms of mouse Oas2 (node center color) and T47D cell lines expressing either mutant (mt) or wild type (wt) forms of mouse Oas2 when treated with Doxycycline (+DOX) (node ring color) for 72 hours. Central map shows network diagram of gene-sets with enrichment values indicated by color scale from red to blue as indicated. Functional role of the clusters of gene sets are shown. Outer panels show box-plots of the GSEA normalised enrichment scores (NES) for the each of the gene-sets in the specified functional clusters. Red crosses indicate positively-enriched gene-sets with FDR<0.05, blue crosses indicate negatively-enriched gene-sets with FDR<0.05 and grey crosses indicate gene-sets that have FDR> = 0.05. (B) Functional groups found common to mouse and human by self organizing maps. (TIF) [file pgen.1007072.s008.tif]

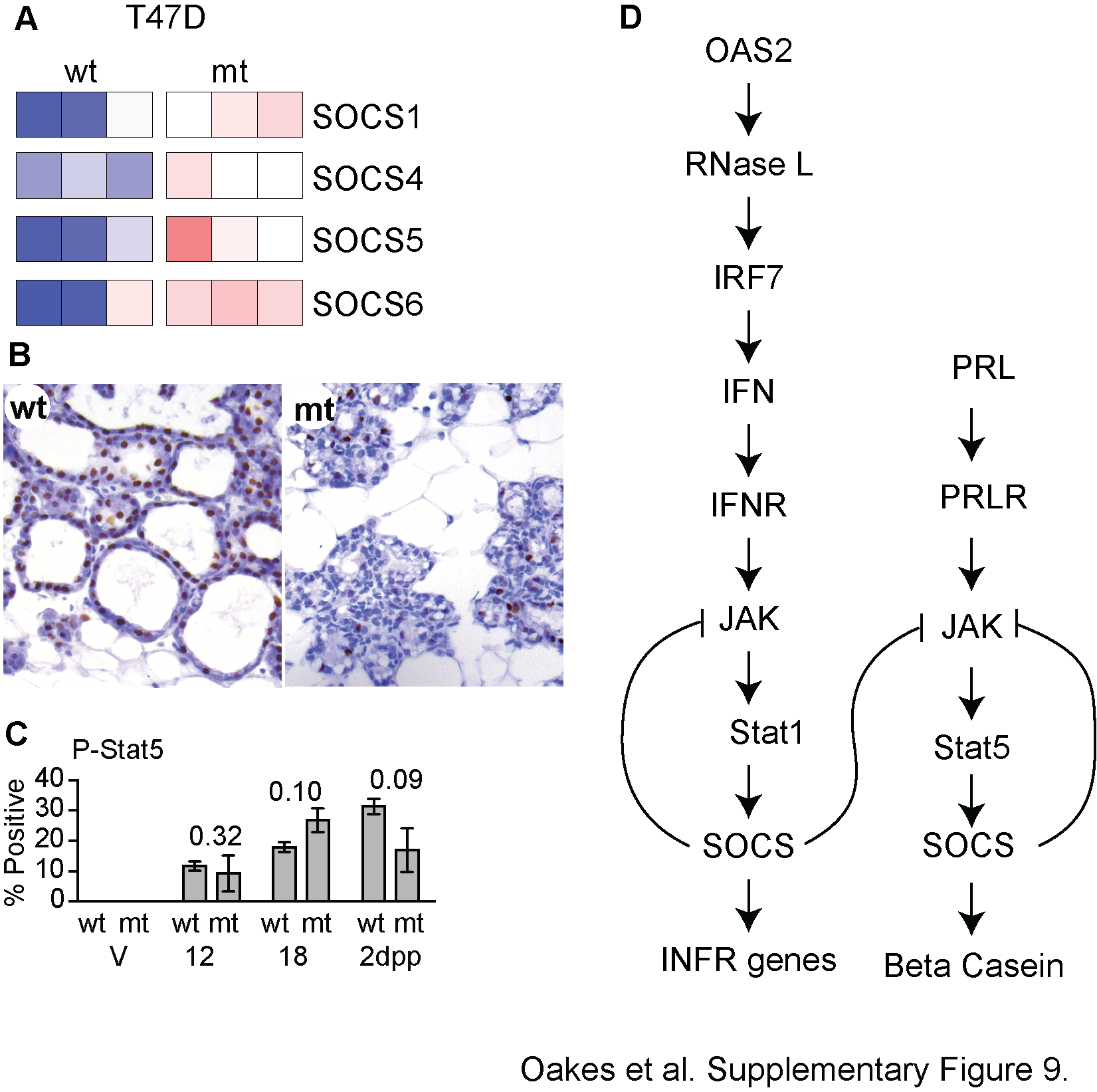

Supplement: S9 Fig — (A) Heatmap extracted from the T47D transcript profiling showing expression changes in SOCS protein gene expression that exceed F value greater than 0.05. (B) Example of immunohistochemistry for STAT5 phosphorylation, which is quantitated in the (C) chart for %positivity. Note this quantification does not capture the increased signal intensity observed in wt glands compared to mt glands. (D)Diagram at the RHS shows the proposed pathway links between interferon and prolactin signaling that may explain the ability of the mutation in OAS2 to prevent lactation. (TIF) [file pgen.1007072.s009.tif]
